# Acellular Pertussis Vaccines Induce CD8^+^ and CD4^+^ Regulatory T Cells That Suppress Protective Tissue‐Resident Memory CD4^+^ T Cells, in Part via IL‐10

**DOI:** 10.1002/eji.202451630

**Published:** 2025-07-09

**Authors:** Caitlín Ní Chasaide, Pauline Schmitt, Béré K. Diallo, Lisa Borkner, Charlotte M. Leane, Seyed Davoud Jazayeri, Sreeram Udayan, Eoin O'Neill, Lucy M. Curham, Barry Moran, Mieszko M. Wilk, Kingston H. G. Mills

**Affiliations:** ^1^ Immune Regulation Research Group School of Biochemistry and Immunology Trinity Biomedical Sciences Institute Trinity College Dublin Dublin Ireland

**Keywords:** pertussis vaccine, Treg cell, T_RM_ cell, Th17 cell, IL‐10

## Abstract

Tissue‐resident memory T (T_RM_) cells play a key role in sustained protective immunity against *Bordetella pertussis* infection of the nasal mucosa. Current alum‐adjuvanted acellular pertussis (aP) vaccines protect against severe pertussis disease but fail to prevent nasal infection with *B. pertussis*. Here we demonstrate that immunization of mice with an aP vaccine failed to generate respiratory T_RM_ cells, but did induce antigen‐specific CD4^+^ Treg cells that expressed Foxp3, CD49b, PD‐1 and LAG‐3, and CD8^+^ Treg cells that expressed CD122, PD‐1, and IL‐10. *B. pertussis*‐specific CD4^+^ and CD8^+^ T cell lines established from aP‐immunized mice expressed the regulatory markers and suppressed activation of Th1 and Th17 cells. Blockade of IL‐10 signaling during aP immunization or *B. pertussis* challenge promoted the induction of IL‐17‐secreting CD4^+^ T_RM_ responses and enhanced bacterial clearance from the nose. Addition of the adjuvant LP‐GMP, comprising TLR2 and STING agonists, to the aP vaccine and delivery by the nasal route promoted the induction of antigen‐specific IL‐17‐producing CD4^+^ T_RM_ cells and enhanced vaccine efficacy. Our findings demonstrate that aP vaccines suppress the induction of protective T_RM_ cells in part through the induction of CD4^+^ and CD8^+^ Treg cells, which can be overcome using a potent adjuvant and delivery of the vaccine intranasally.

## Graphical abstract

The authors demonstrate that current aP vaccines induce IL‐10‐secreting CD4^+^ and CD8^+^ Treg cells that suppress IL‐17‐secreting respiratory tissue‐resident memory (T_RM_) cells, and thereby fail to prevent nasal infection with *B. pertussis*. Blocking IL‐10R *in vivo* or adding a novel adjuvant LP‐GMP and intranasal delivery reverses suppression and enhances vaccine efficacy. Created with BioRender.com.

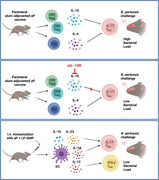

**Abbreviations**
aP vaccine: acellular pertussis vaccine
APC: antigen‐presenting cell
BCG: Bacille Calmette‐Guérin
CFU: colony‐forming unit
DC: dendritic cell
dLN: draining lymph node
FHA: filamentous hemagglutinin
Foxp3: forkhead box P3 transcription factor
i.m.: intramuscular
i.n.: intranasal
i.p.: intraperitoneal
LAG‐3: lymphocyte activation gene 3
LN: lymph node
PAMPs: pathogen‐associated molecular patterns
PD‐1: programmed cell death protein 1
PECs: peritoneal exudate cells
PRN: pertactin
RORγt: retinoic acid‐related orphan receptor‐γt
sBp: sonicated *Bordetella pertussis*
STING: stimulator of interferon genes
T‐bet: T‐box transcription factor
T_eff_
: effector T cells
Tr1 cell: regulatory type‐1 cell
Treg cell: regulatory T cell
T_RM_ cells: tissue‐resident memory T cells
wP vaccine: whole cell pertussis vaccine

## 1 Introduction

Whooping cough (pertussis) is a respiratory disease caused by the Gram‐negative bacterium *Bordetella pertussis*, which is most severe and potentially fatal in infants [1]. Despite high vaccination coverage with the acellular pertussis (aP) vaccine in high‐income countries, cyclical epidemics of pertussis occur every 2–5 years, with significant outbreaks in Europe and the US in the last year [2, 3, 4]. Various factors, including waning of aP vaccine‐induced immunity, vaccine‐driven mutation of *B. pertussis*, and enhanced detection techniques, may contribute to the resurgence of pertussis [5, 6, 7]. However, a significant contributory factor is the inability of aP vaccines to generate sterilizing immunity against *B. pertussis* infection in the nasal mucosa [8, 9]. Consequently, aP vaccines do not prevent transmission of the bacteria [10], leaving very young infants susceptible to pertussis and possible death before they have completed their vaccination program.

Natural infection with *B. pertussis* and immunization with wP vaccines prevent pertussis disease and infection of the respiratory tract [11, 12, 13], and this is associated with the recruitment of IL‐17^+^ and IFN‐γ^+^ CD4^+^ tissue‐resident memory T (T_RM_) cells to respiratory tissues in mice [8] and humans [14]. CD4^+^ T_RM_ cells express CD44 and CD69, with or without CD103, lack CD62L expression, and reside in the respiratory tissues following infection, poised to respond rapidly upon re‐exposure [15, 16]. IL‐17‐producing CD4^+^ T_RM_ cells, which are recruited to the respiratory tissues during *B. pertussis* infection and following immunization with wP vaccines, play an indispensable role in the clearance of *B. pertussis* from the nasal mucosa, through recruitment of Siglec‐F^+^ neutrophils [8, 17, 18, 19]. However, current aP vaccines fail to induce IL‐17‐ or IFN‐γ‐secreting CD4^+^ T_RM_ cells in the respiratory tissue of mice [8] or humans [14].

Studies in baboons have shown that commercial alum‐adjuvanted aP vaccines are effective at preventing severe pertussis disease, but do not prevent nasopharyngeal colonization with *B. pertussis*, or bacterial transmission to naïve animals [9]. Although aP vaccines induce potent antibody and Th2‐skewed responses, studies in mice have shown that they fail to induce Th17 responses, and may suppress the expansion of IL‐17^+^ T_RM_ cells in the respiratory tissues following *B. pertussis* challenge [8, 18]. Furthermore, immunization with the aP vaccine prolongs infection in the nasal mucosa following the challenge of BALB/c mice with *B. pertussis* [18].

Current aP vaccines are formulated with 2–5 *B. pertussis* antigens, including filamentous hemagglutinin (FHA) [20]. FHA promotes IL‐10 production by dendritic cells (DCs) and macrophages and directs naïve T cells to differentiate into IL‐10‐secreting regulatory type‐1 (Tr1) cells during infection with *B. pertussis* [21, 22, 23]. IL‐10 is an immunosuppressive cytokine produced by various cell types, including macrophages, DCs, and regulatory T (Treg) cells [24]. Alum, the adjuvant used for all licensed aP vaccines, has been shown to induce IL‐10, which leads to suppression of antigen‐specific Th1 responses [25]. The related bacterial species *Bordetella bronchiseptica* evades immune responses in infected mice by inducing IL‐10, which inhibits IFN‐γ production, resulting in prolonged infection of the lung [26, 27]. IL‐10 has been shown to facilitate persistence of *Staphylococcus aureus* in the nasal cavity of mice by suppressing nasal IL‐17^+^ and IL‐22^+^ T cell responses [28]. Furthermore, IL‐10 has been implicated in dampening Th1 responses induced by the Bacille Calmette‐Guérin (BCG) vaccine and Th17 responses induced by *S. aureus* vaccines [29, 30, 31].

Attempts to improve the efficacy of aP vaccines have focused on the use of novel adjuvants, including pathogen‐associated molecular patterns (PAMPs) [32, 33, 34, 35, 36]. Current aP vaccines are delivered parenterally, and recent studies have demonstrated that the intranasal (i.n.) route of immunization is more effective for the induction of mucosal antibodies and T cells [37]. We have reported that i.n. immunization of mice with an experimental aP vaccine formulated with the adjuvant LP‐GMP, comprising the *B. pertussis*‐derived TLR2 agonist, LP1569, combined with the stimulator of interferon genes (STING) agonist, cyclic‐di‐GMP, led to sustained protective immunity in the nasal cavity in mice, which correlated with IL‐17^+^ CD4^+^ T_RM_ cells in the nose [38].

In this study, we demonstrated that current alum‐adjuvanted aP vaccines induce antigen‐specific IL‐10 and CD4^+^ and CD8^+^ Treg cells. Blockade of IL‐10 signaling at the time of aP immunization, or *B. pertussis* challenge in aP‐immunized mice, attenuated the suppression of T_RM_ responses and enhanced protection against infection in the nasal mucosa. Finally, the addition of LP‐GMP to the alum‐adjuvanted aP vaccine and delivery by the nasal route overcame the suppressive effects of the aP vaccine, allowed induction of IL‐17‐secreting T_RM_ cells, and enhanced vaccine efficacy.

## 2 Results

### 2.1 aP Vaccine Induces CD4^+^ and CD8^+^ Treg Cells That Suppress *B. pertussis*‐specific IL‐17 and IFN‐γ Production Through IL‐10

Current aP vaccines do not prevent nasal infection with *B. pertussis*, and this has been linked to their selective induction of Th2 cells and suppression of respiratory T_RM_ cells [8, 18]. We had previously demonstrated that infection with *B. pertussis* induces IL‐10‐secreting CD4^+^ Treg cells [21]. Here, we examined the hypothesis that aP vaccines may also promote the induction of Treg cells that produce IL‐10 and that this may contribute to their immunosuppressive effects.

**FIGURE 1:**
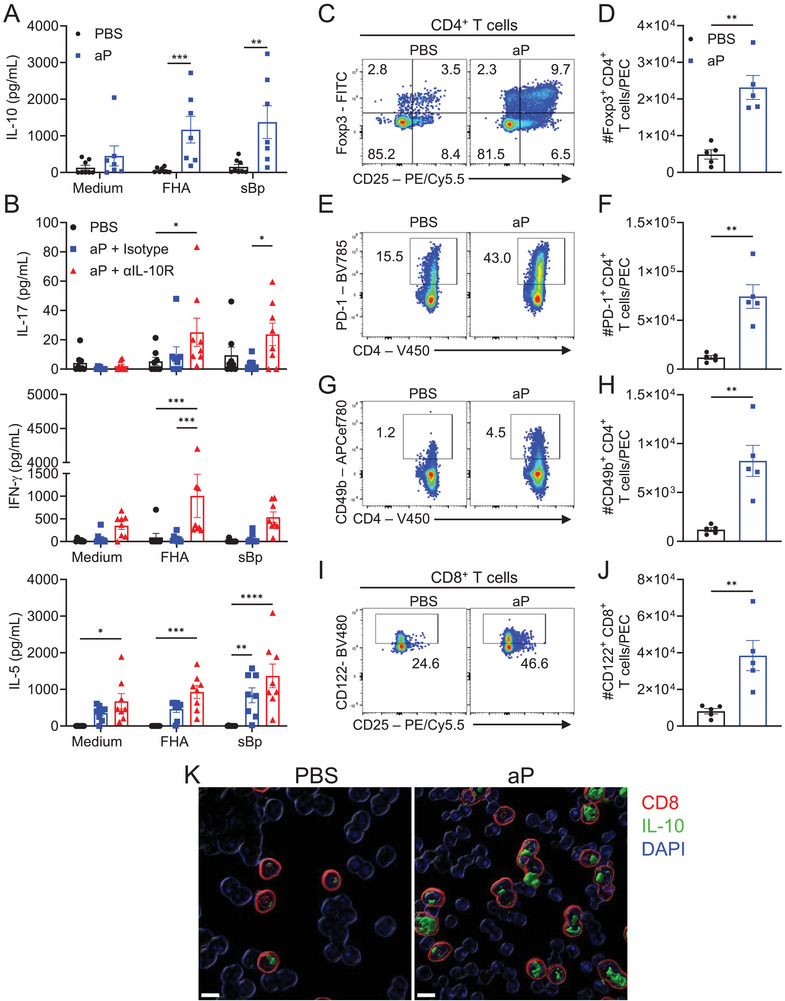
Alum‐adjuvanted aP vaccine induces IL‐10, which inhibits Th1 and Th17 responses. (A) Mice were immunized i.p. with Infanrix twice (0 and 28 days). Three days after the second immunization, mediastinal LN cells (dLN; 2 × 10^5^ cells/well) were cultured with FHA (2 µg/mL), sBp (1 µg/mL), or medium alone. After 3 days, the concentrations of IL‐10 in culture supernatants were quantified by ELISA. Data shown are ±SEM (*n* = 7–8 mice per group), with each symbol representing an individual mouse, data pooled from two independent experiments. **p < 0.01, ***p < 0.001 by Mann–Whitney test. (B) Mice were immunized i.p. at 0 and 28 days with Infanrix and treated with anti‐IL‐10R or isotype control antibody (200 µg/mouse; i.p.) one day prior to and 4 h after immunization. Two weeks after the second immunization, PECs were harvested and cultured (2 × 10^5^ cells/well) with FHA (2 µg/mL), sBp (1 µg/mL), or medium alone. After 72 h, concentrations of IL‐17, IFN‐γ, and IL‐5 in cell culture supernatants were quantified by ELISA. Data shown are mean ± SEM (*n* = 8 mice per group) with each symbol representing a triplicate culture for an individual mouse; data are pooled from two independent experiments. *p < 0.05, **p < 0.01, ***p < 0.001, ****p < 0.0001 by two‐way ANOVA with Tukey's post‐test. (C–K) Mice were immunized i.p. with the aP vaccine (Infanrix; 1/50 human dose) on days 0 and 28, or with PBS as control. Seven days after second immunization, PECs, mediastinal dLNs, and spleens were harvested. (C–J) PECs were stained with cell surface markers CD11b, CD19, CD3, CD4, CD8, CD49b, CD122 and PD‐1 and intranuclearly for Foxp3. Representative flow cytometry plots (C) and mean absolute numbers (D) of Foxp3^+^CD4^+^CD19^−^CD11b^−^ cells. Representative flow cytometry plots (E) and mean absolute numbers (F) of PD‐1^+^CD4^+^CD19^−^CD11b^−^ cells. Representative flow cytometry plots (G) and mean absolute numbers (H) of CD49b^+^CD4^+^CD19^−^CD11b^−^ cells. Representative flow cytometry plots (I) and mean absolute numbers (J) of CD122^+^CD8^+^CD19^−^CD11b^−^ cells. Data shown are mean ±SEM (*n* = 5 mice per group), from one experiment. **p < 0.01 by Mann–Whitney test. (K) Imaris reconstruction of IL‐10^+^ CD8^+^ T cells in spleen and dLN, following 20 h co‐culture of cells, at a 1:1 ratio (8 × 10^5^ cells/well), with FHA (2 µg/mL) and anti‐CD49d and anti‐CD28 (both at 1 µg/mL). Brefeldin A (5 µg/mL) and monensin (1 µg/mL) were added for the final 4 h, prior to immunocytochemistry staining. Representative image from five mice per group in one experiment. Scale bars = 8 µm.

We first examined the induction of immune response following immunization of mice with an aP vaccine via the intraperitoneal (i.p.) route, which facilitated easy access to locally produced immune cells via peritoneal lavage. We detected significant IL‐10 in draining lymph node (dLN) cells from mice immunized twice with the aP vaccine following 3 days of stimulation with the antigen FHA or sonicated *B. pertussis* (sBp) (Figure 1A). We next examined the effect of blocking IL‐10 signaling *in vivo* on antigen‐specific T cell responses induced with the aP vaccine. Immunization of mice with the aP vaccine (and treatment with an isotype control antibody) induced antigen‐specific Th2, but not Th1 or Th17, responses (Figure 1B). IL‐5 was detected in supernatants of peritoneal exudate cells (PECs) stimulated with FHA or sBp, whereas the concentration of IL‐17 and IFN‐γ was not significantly greater than that detected in PBS‐immunized control mice (Figure 1B). However, injection of an anti‐IL‐10R antibody at the time of immunization with the aP vaccine significantly enhanced IL‐17, IFN‐γ, and IL‐5 production by PECs, stimulated with FHA or sBp (Figure 1B).

We next examined the possible induction of Treg cells following immunization of mice with an aP vaccine. Flow cytometry analysis of PECs in mice immunized with the aP vaccine compared with mice immunized with PBS revealed significant expansion of CD4^+^ T cells that expressed Foxp3 and PD‐1 and the Tr1 markers, CD49b and LAG‐3 (Figure 1C–H; Figure S1A,B). In contrast, the Th1 cell transcription factor T‐bet was not enhanced in aP‐immunized mice (Figure S2A,B). Although expression of RORγt was also increased, a large proportion (68.8%) of RORγt^+^ CD4^+^ T cells in PEC of aP‐immunized mice co‐expressed Foxp3 (Figure S2C–F). Foxp3^+^RORγt^+^ CD4^+^ T cells have been shown to have a regulatory function, differing from pro‐inflammatory Foxp3^−^RORγt^+^ Th17 cells [39, 40]. Immunization with the aP vaccine also induced CD8^+^ T cells that expressed CD122 (Figure 1I,J), a marker associated with CD8^+^ Treg cells. There was also a small expansion of CD8^+^Foxp3^+^ T cells (Figure S1C,D). We also observed an induction of Foxp3^+^ and CD49b^+^ CD4^+^ T cells (Figure S3A–D) and CD8^+^ T cells, which co‐expressed CD122 and PD‐1 (Figure S3E–H), in mediastinal dLNs of mice immunized with the aP vaccine. PD‐1^+^CD122^+^ have been shown partly to mediate their regulatory function through the production of IL‐10 [41, 42]. We showed by immunocytochemistry analysis that *B. pertussis*‐specific CD8^+^ T cells from aP‐immunized mice secreted IL‐10; substantial IL‐10 expression is evident in proliferating CD8^+^ T cells from aP‐immunized but not from control mice (Figure 1K).

These data are consistent with previous studies showing that commercial aP vaccines induce Th2 cells but do not generate *B. pertussis*‐specific Th1 and Th17 cells, and extend these findings to demonstrate that antigen‐specific IL‐10 and CD4^+^ and CD8^+^ Tregs are also induced following immunization with the aP vaccine and that these may suppress induction of Th1 and Th17 cells.

**FIGURE 2:**
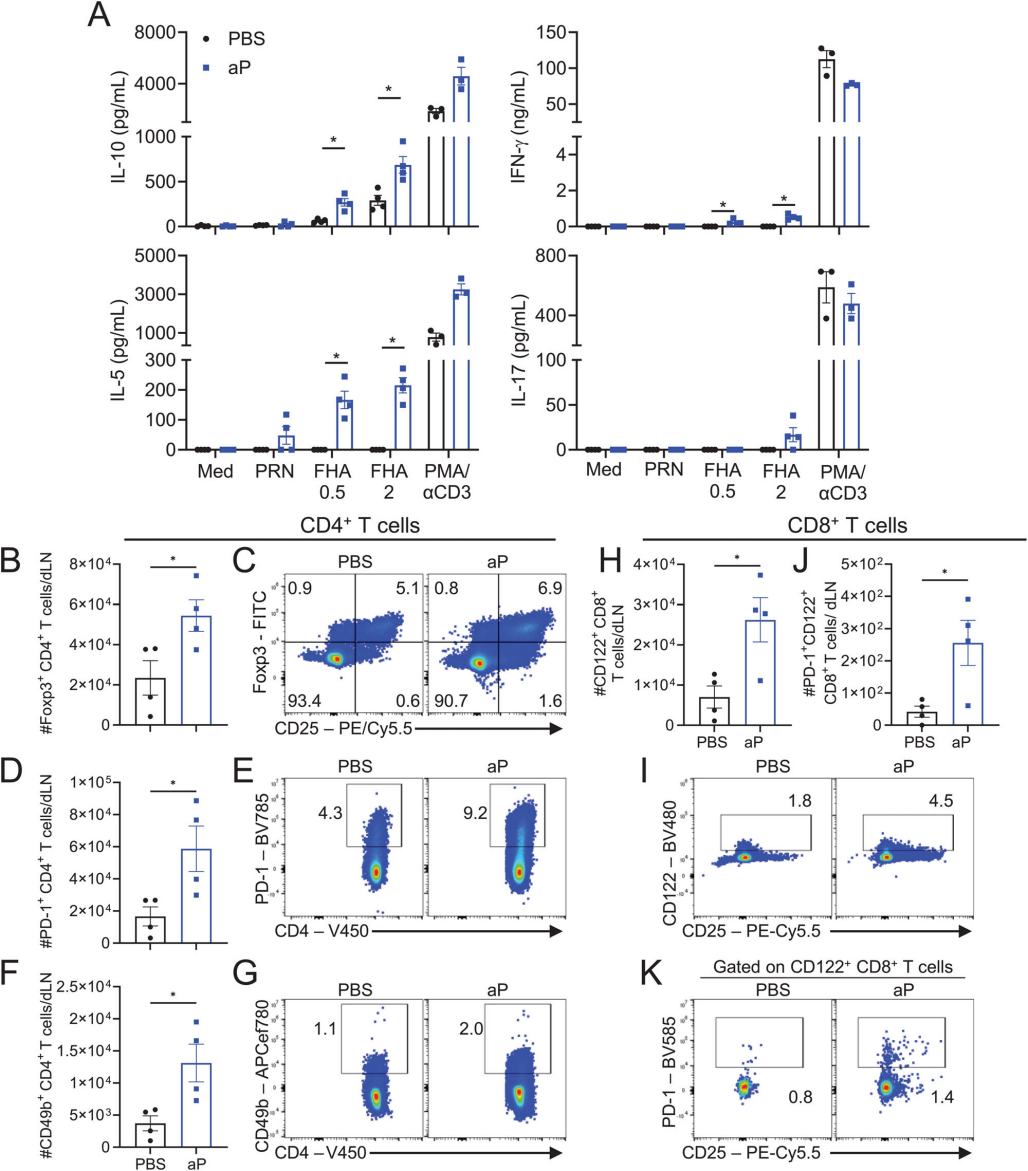
The alum‐adjuvanted aP vaccine induces antigen‐specific Treg cells and IL‐10 when administered by i.m. route. Mice were immunized i.m. with aP vaccine (Boostrix; 1/10 human dose) or with PBS as control on days 0 and 28. On day 35, inguinal and popliteal LNs (dLNs) and spleens were harvested. (A) Spleen and dLN cells were co‐cultured at a 1:1 ratio (4 × 10^5^ cells/well) with PRN (1 µg/mL), FHA (0.5 or 2 µg/mL), PMA and anti‐CD3 (αCD3), or medium alone for 72 h. Concentrations of IL‐10, IFN‐γ, IL‐5, and IL‐17 in supernatants were quantified by ELISA. Data shown are mean ± SEM (*n* = 4 mice per group) with each symbol representing triplicate culture for an individual mouse (For PMA/αCD3 stimulation condition, data shown are mean ±SEM (*n* = 3 mice per group) with each symbol representing single‐well culture for an individual mouse), representative of two independent experiments. *p < 0.05 by Mann–Whitney test. (B–K) dLN cells were stained with Treg surface markers and intranuclearly for Foxp3 for flow cytometric analysis. Mean absolute cell numbers and representative flow cytometry plots of Foxp3^+^CD4^+^CD19^−^CD11b^−^ cells (B, C), PD‐1^+^CD4^+^CD19^−^CD11b^−^ cells (D, E), CD49b^+^CD4^+^CD19^−^CD11b^−^ cells (F, G), CD122^+^CD8^+^CD19^−^CD11b^−^ cells (H, I) and PD‐1^+^CD122^+^CD8^+^CD19^−^CD11b^−^ cells (J, K). Data shown are mean ±SEM (*n* = 4 mice per group), with each symbol representing an individual mouse, from one experiment. *p < 0.05, by unpaired *t*‐test.

To further investigate the antigen‐specific IL‐10 and CD4^+^ and CD8^+^ Treg cells induced by the aP vaccine, mice were immunized with the DTaP vaccine Infanrix or the TdaP vaccine Boostrix by the intramuscular (i.m.) route, the route used for human vaccination. We found that FHA‐specific T cells in the dLNs and spleen from mice with Boostrix secreted a significant concentration of IL‐10 and IL‐5, low concentrations of IFN‐γ, but no significant IL‐17 (Figure 2A). Similarly, dLN and spleen cells from mice immunized i.m. with Infanrix secreted significant concentrations of FHA‐specific IL‐10 and IL‐5, but very low concentrations of IFN‐γ and IL‐17 following *in vitro* stimulation with FHA (Figure S4). Responses to the aP vaccine antigen, pertactin (PRN), were weak or undetectable. These findings suggest that aP vaccines promote the induction of FHA‐specific T cells that secrete IL‐10. Flow cytometry analysis of dLN cells from mice immunized with the aP vaccine revealed a significant expansion of CD4^+^ and CD8^+^ T cells that expressed regulatory markers. There was a significant increase in the absolute number of CD4^+^ T cells expressing Foxp3, PD‐1, and CD49b in aP‐immunized compared with control mice (Figure 2B–G). Furthermore, there was an expansion of CD8^+^ T cells that expressed CD122 or that co‐expressed PD‐1 and CD122 in mice immunized with the aP vaccine (Figure 2H–K). We confirmed by immunocytochemistry analysis that *B. pertussis*‐specific CD8^+^ T cells from mice immunized i.m. with the aP vaccine secreted IL‐10 and proliferated in response to stimulation with FHA (Figure S5).

**FIGURE 3:**
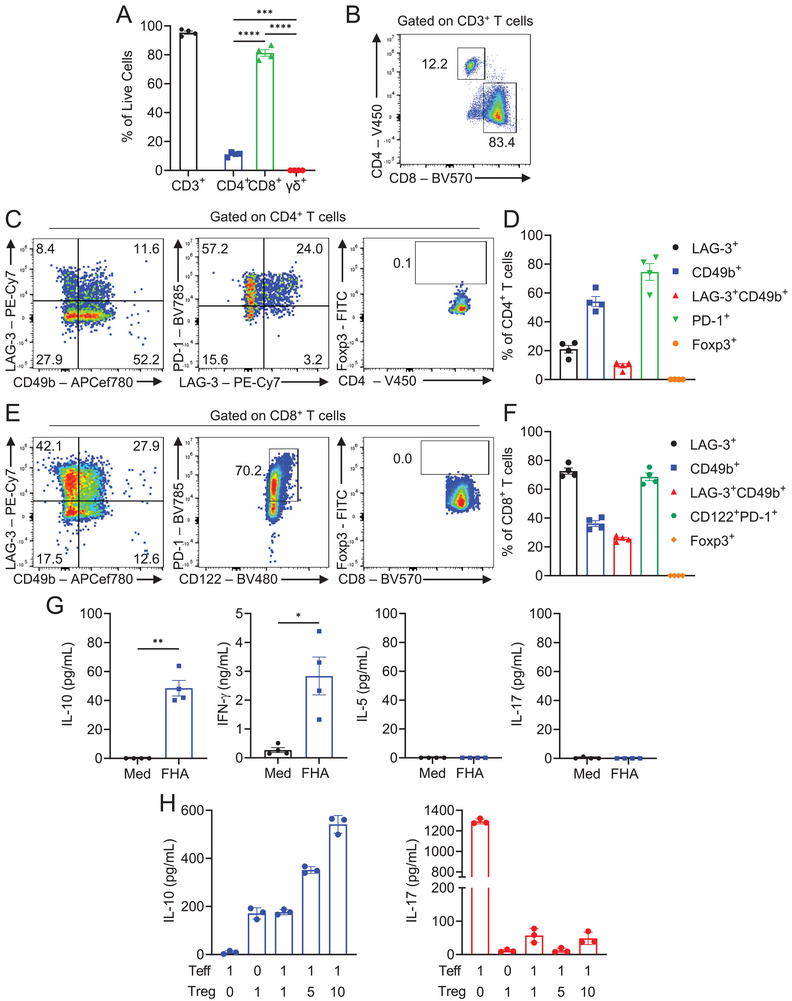
T cell lines established from aP‐immunized mice express Treg markers and suppress antigen‐specific Th17 cells. Mice were immunized i.m. with aP vaccine (Boostrix; 1/10 human dose) on days 0 and 28. On day 35, T cell lines were generated by culturing cells with FHA in the presence of IL‐2 and IL‐15. T cells were stained with antibodies specific for Treg cell surface markers and analyzed by flow cytometry. Mean frequency of CD3^+^, CD4^+^, CD8^+^, and γδ^+^ T cells (A), and representative flow cytometry plots for CD4 versus CD8 (B). Representative flow cytometry plots (C) and mean frequencies (D) of LAG‐3^+^, CD49b^+^, LAG‐3^+^CD49b^+^, PD‐1^+^, and Foxp3^+^ CD4^+^ T cells (all pregated on CD4^+^CD19^−^CD11b^−^CD45.2^+^ cells). Representative flow cytometry plots (E) and mean frequencies (F) of LAG‐3^+^, CD49b^+^, LAG‐3^+^CD49b^+^, CD122^+^PD‐1^+^, and Foxp3^+^ CD8^+^ T cells (all pregated on CD8^+^CD19^−^CD11b^−^CD45.2^+^ cells). (G) Cells from an FHA‐specific T cell line were stimulated with FHA (2 µg/mL) or medium only in the presence of splenic irradiated APCs for 72 h. Concentrations of IL‐10, IFN‐γ, IL‐17, and IL‐5 were quantified by ELISA. Data shown are mean ± SEM (*n* = 4 mice, biological replicates), with each symbol representing a T cell line established from an individual mouse, representative of two independent experiments. ***p < 0.001, ****p < 0.0001 by one‐way ANOVA with Tukey's post‐test (**A**) or *p < 0.05, **p < 0.01 by paired *t*‐test (G). (H) CD4^+^ T_eff_ cells isolated from LNs (cervical, axillary, brachial) and lungs of convalescent mice were co‐cultured (0.5 × 10^5^ cells/well) with Treg cell lines (0.5 × 10^5^, 2.5 × 10^5^, 5 × 10^5^ cells/well), irradiated APCs (5 × 10^5^ cells/well) and FHA (2 µg/mL). After 4 days, IL‐17 and IL‐10 concentrations in culture supernatants were quantified by ELISA. Data shown are mean ± SD (pooled from four individual T cell lines shown in A–G) of triplicate cell culture (technical replicates), representative of two independent experiments.

To provide further evidence that aP vaccines induced antigen‐specific T cells with a regulatory phenotype that secrete IL‐10, FHA‐ and *B. pertussis*‐specific T cell lines were generated from mice immunized with the aP vaccine, by culturing dLN and spleen cells with FHA or sBp in the presence of IL‐2, or IL‐2 in combination with IL‐15. dLN and spleen cells from mice immunized with an aP vaccine cultured in the absence of antigen did not survive, whereas dLN and spleen cells cultured with FHA or sBp generated antigen‐specific T cell lines. Phenotypic characterization using multiparameter spectral flow cytometry revealed that FHA and *B. pertussis*‐specific T cell lines were 95% and 91% CD3^+^ T cells, respectively, which were predominantly CD8^+^ T cells, with a smaller population of CD4^+^ T cells (Figure 3A,B; Figure S6A,B). However, the frequency of CD8^+^ T cells was lower in T cell lines stimulated with FHA or sBp in the absence of IL‐15 (data not shown); IL‐15 is required for the expansion of CD8^+^ T cells [43]. Both the CD4^+^ and CD8^+^ T cells in the FHA‐specific T cell lines expressed markers of peripheral Treg cells. The majority of CD4^+^ T cells expressed PD‐1, and a proportion also expressed LAG‐3 and CD49b, markers of Tr1 cells (Figure 3C,D). The CD8^+^ T cells in the T cell line included a substantial population of LAG‐3^+^CD49b^+^ ‘Tr‐1‐like’ CD8^+^ T cells (Figure 3E,F). A substantial number of the CD8^+^ T cells co‐expressed CD122 and PD‐1 (Figure 3E,F). Similar CD4^+^ and CD8^+^ Treg cell phenotypes were observed for T cell lines generated with sBp as the antigen (Figure S6C–F). Neither CD4^+^ nor CD8^+^ T cells in the antigen‐specific T cell line expressed Foxp3, which is consistent with the expansion of peripheral Treg cells that normally do not express Foxp3.

FHA‐specific Treg cell lines from aP‐immunized mice produced IL‐10 and IFN‐γ, but no IL‐5 or IL‐17, following restimulation with FHA (Figure 3G). The *B. pertussis*‐specific Treg cell lines produced significant antigen‐specific IL‐10 and IFN‐γ, but no significant IL‐17 (Figure S6G).

We examined the suppressive activity of the Treg cell line by co‐culture with antigen and CD4^+^ T cells isolated from lungs and LNs of convalescent mice. The FHA‐specific Treg cell lines suppressed antigen‐specific IL‐17 production from the convalescent CD4^+^ effector T (T_eff_) cells (Figure 3H). Furthermore, FHA‐specific T cell lines produced IL‐10 in a cell concentration‐dependent manner (Figure 3H). A similar suppressive function was observed for the *B. pertussis*‐specific Treg cell line (Figure S6H).

Collectively, these data suggest that immunization of mice with an aP vaccine induces CD4^+^ and CD8^+^ Treg cells that suppress protective *B. pertussis*‐specific Th17 responses.

### 2.2 aP Vaccine‐Induced IL‐10 Inhibits Protective Nasal T_RM_ Responses

**FIGURE 4:**
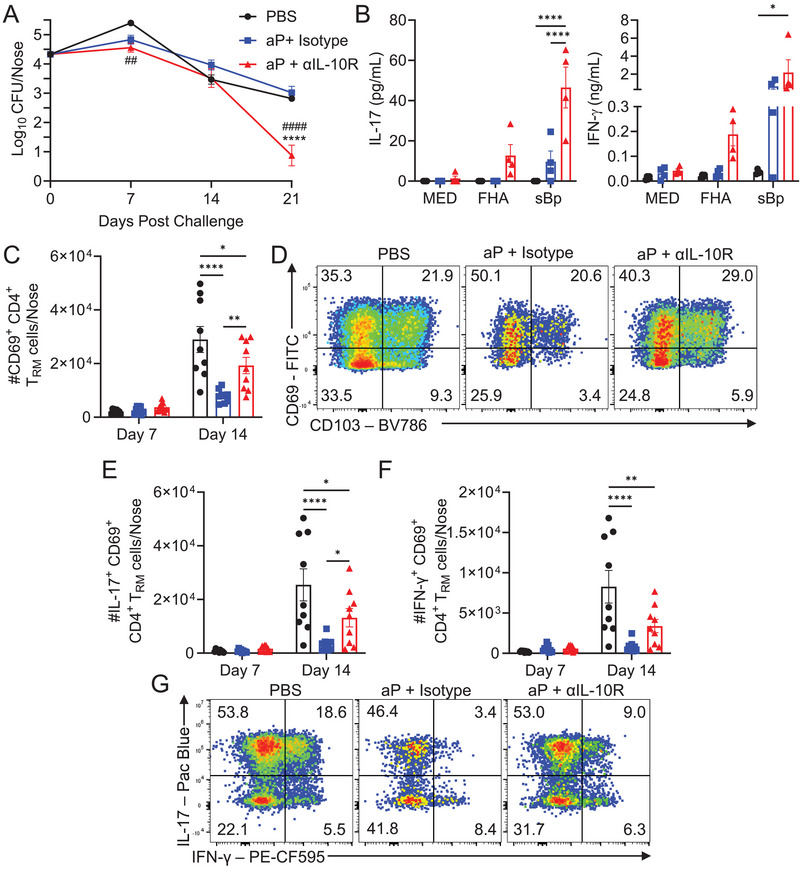
Blockade of IL‐10R at the time of aP immunization enhances *B. pertussis* clearance and ameliorates T_RM_ cell suppression in the nose. Mice were immunized i.p. at days 0 and 28 with aP vaccine (Infanrix; 1/50 human dose) and treated i.p. with an αIL‐10R or isotype control antibody, 1 day prior to and 3 days after immunizations or mice were immunized with PBS as a control. (A) Mice were aerosol challenged with live *B. pertussis* 14 days after the booster immunization, and bacterial CFUs were assessed in nasal tissue at 2–16 h (day 0) and on days 7, 14, and 21 post challenge. Data are mean ± SEM (*n* = 7–9 mice per group, per time‐point), and data are pooled from two independent experiments. ****p < 0.0001 aP + αIL‐10R versus aP + Isotype, ##*p* < 0.01, ####*p* < 0.0001 aP + αIL‐10R versus PBS, by two‐way ANOVA with Tukey's post‐test. (B) On the day of challenge, purified lung immune cells were co‐cultured (in triplicate) with irradiated APCs, and FHA (2 µg/mL), sBp (5 µg/mL), or medium for 72 h. IL‐17 and IFN‐γ concentrations were quantified by ELISA. (C–G) On days 7 and 14 post‐*B. pertussis* challenge, mice were injected i.v. with αCD45 antibody 10 min prior to euthanasia, and nasal tissue cells were stained with antibodies specific for T_RM_ cells, or cells were stimulated with PMA and ionomycin for 4 h, prior to intracellular cytokine staining (ICS) and flow cytometric analysis. Mean absolute number of CD69^+^ CD4^+^ T_RM_ cells (C) with representative flow cytometry plots for day 14 (D). Mean absolute number of IL‐17^+^ (E) and IFN‐γ^+^ (F) CD4^+^ T_RM_ cells with representative flow cytometry plots for day 14 (G). Data are mean ±SEM (*n* = 4 (B) or *n* = 8–9 (C–G) mice per group, per time‐point), with each symbol representing an individual mouse. All data are pooled or representative of two independent experiments. The control groups in one of the two experiments were shared with those in Figure 5. *p < 0.05, **p < 0.01 ****p < 0.0001 by two‐way ANOVA with Tukey's post‐test.

We next examined the effect of blocking IL‐10 signaling during immunization or *B. pertussis* challenge on the induction of protective T cells in the respiratory tract. Treatment of mice with an anti‐IL‐10R antibody at the time of immunization with the aP vaccine resulted in significantly enhanced clearance of *B. pertussis* from the nasal mucosa following aerosol challenge with *B. pertussis* (Figure 4A). Blockade of IL‐10R at the time of aP immunization significantly enhanced *B. pertussis*‐specific IL‐17 and IFN‐γ production by lung immune cells on the day of challenge (Figure 4B).

CD69^+^ CD4^+^ T_RM_ cells were detected in the nasal tissue 14 days after aerosol challenge of naïve mice with *B. pertussis* (Figure 4C, D). However, the accumulation of CD69^+^CD4^+^ T_RM_ cells was significantly suppressed in mice immunized with the aP vaccine and treated with isotype control antibody. Blockade of IL‐10 signaling *in vivo* reversed the suppression of CD4^+^ T_RM_ cells (Figure 4C,D). IL‐17‐ and IFN‐γ‐secreting CD4^+^ T_RM_ cells were detected in the nasal tissue 14 days after aerosol challenge of naïve mice with *B. pertussis* (Figure 4E–G). However, IL‐17^+^ and IFN‐γ^+^ T_RM_ cells were suppressed in mice immunized with the aP vaccine and treated with an isotype control antibody. Blockade of IL‐10 signaling at the time of immunization significantly reversed the suppression of IL‐17^+^ T_RM_ cells (Figure 4E,G) and partially attenuated the suppression of IFN‐γ^+^ T_RM_ cells (Figure 4F,G). These data demonstrate that immunization with the aP vaccine suppresses the accumulation of CD4^+^ T_RM_ cells in the nasal tissue and that this is mediated in part by the induction of IL‐10.

**FIGURE 5:**
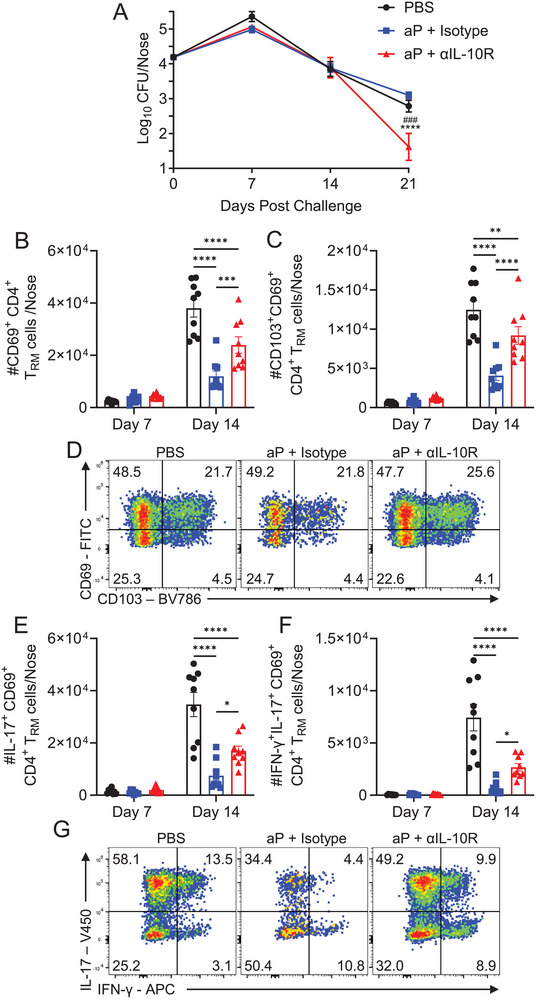
Blockade of IL‐10R at the time of *B. pertussis* challenge enhances bacterial clearance and reverses T_RM_ suppression in nasal mucosa in aP‐immunized mice. Mice were immunized i.p. at days 0 and 28 with aP vaccine (Infanrix; 1/50 human dose) or PBS. Mice were aerosol challenged with live *B. pertussis* 2 weeks after boost and were treated i.p. with αIL‐10R or isotype control antibody, 1 day and 4 h prior to and on days 4, 8, 12, and 16 after challenge. (A) Bacterial CFUs were enumerated in nasal tissue at 16 h (day 0) and on days 7, 14, and 21 post challenge. Data are mean ±SEM (*n* = 8–9 mice per group, per time‐point) and are pooled from two independent experiments. ****p < 0.0001 vs. aP + Isotype, ###*p*<0.001 vs PBS, by two‐way ANOVA with Šidák's post‐test. (B–G) On days 7 and 14, mice were injected i.v. with αCD45 antibody 10 min prior to euthanasia. Nasal cells were stained with antibodies specific for T_RM_ cell surface markers, or cells were stimulated with PMA and ionomycin for 4 h, prior to ICS and flow cytometric analysis. Mean absolute numbers of CD69^+^ (B) and CD103^+^CD69^+^ (C) CD4^+^ T_RM_ cells, with representative flow cytometry plots for day 14 (D). Mean absolute cell number of IL‐17^+^ (**E**) and IFN‐γ^+^IL‐17^+^ (F) CD4^+^ T_RM_ cells with representative flow cytometry plots for day 14 (G). Data shown are mean ±SEM (*n* = 8–9 mice per group, per time‐point), with each symbol representing an individual mouse. All data are pooled from two independent experiments. The control groups in one of the two experiments were shared with those in Figure 4. *p < 0.05, **p < 0.01, ***p < 0.001, ****p < 0.0001 by two‐way ANOVA with Tukey's post‐test.

We next examined the effect of blocking the IL‐10R during *B. pertussis* challenge of mice immunized with the aP vaccine. Treatment with anti‐IL‐10R 1 day and 4 h prior to and on days 4, 8, 12, and 16 after *B. pertussis* challenge of mice immunized with the aP vaccine enhanced clearance of the bacteria; the CFU counts in the nasal tissue were significantly lower 21 days after *B. pertussis* challenge in aP‐immunized mice treated with anti‐IL‐10R antibody compared with an isotype control antibody (Figure 5A). Blockade of IL‐10 signaling significantly reversed aP vaccine‐induced suppression of CD69^+^CD4^+^ and CD69^+^CD103^+^ CD4^+^ T_RM_ cells in the nasal tissue on day 14 post challenge (Figure 5B–D). Furthermore, blockade of the IL‐10R significantly reversed the suppression of IL‐17^+^ and IL‐17^+^IFN‐γ^+^ CD4^+^ T_RM_ cells in mice immunized with the aP vaccine (Figure 5E–G). We also demonstrated that immunization with that aP vaccine suppressed the accumulation of CD69^+^CD8^+^, CD69^+^CD103^+^CD8^+^ T_RM_ cells and IFN‐γ^+^CD8^+^ T_RM_ cells in the nasal tissue 14 days after challenge with *B. pertussis* and that this was reversed by blockade of the IL‐10R (Figure S8A–F). IL‐17^+^CD8^+^ T_RM_ cells were not expanded by immunization with the aP vaccine (Figure S8D,F).

These data demonstrate that aP vaccines promote the induction of IL‐10‐secreting T cells that suppress activation of Th17 cells and the accumulation of IL‐17^+^ CD4^+^ T_RM_ cells in the respiratory tissue and thereby fail to confer protection against nasal infection with *B. pertussis*.

### 2.3 Addition of a Potent Mucosal Adjuvant LP‐GMP and Delivery by the Nasal Route Reverses Suppression of Th17 Cells and Enhances the Efficacy of a Commercial aP Vaccine

Delivery of vaccines by the nasal route, especially with powerful mucosal adjuvants, is known to promote the induction of T_RM_ cells. We have reported that a nasally delivered experimental aP vaccine comprising FHA, PRN, and recombinant pertussis toxin formulated with the novel adjuvant LP‐GMP generated robust nasal IL‐17^+^ T_RM_ responses and sustained protective immunity in the nasal mucosa [38]. Here, we assessed whether suppression of protective T_RM_ responses induced by the commercial alum‐adjuvanted aP vaccine could be overcome by the addition of a mucosal adjuvant LP‐GMP and delivery of the vaccine by the i.n. route.

**FIGURE 6:**
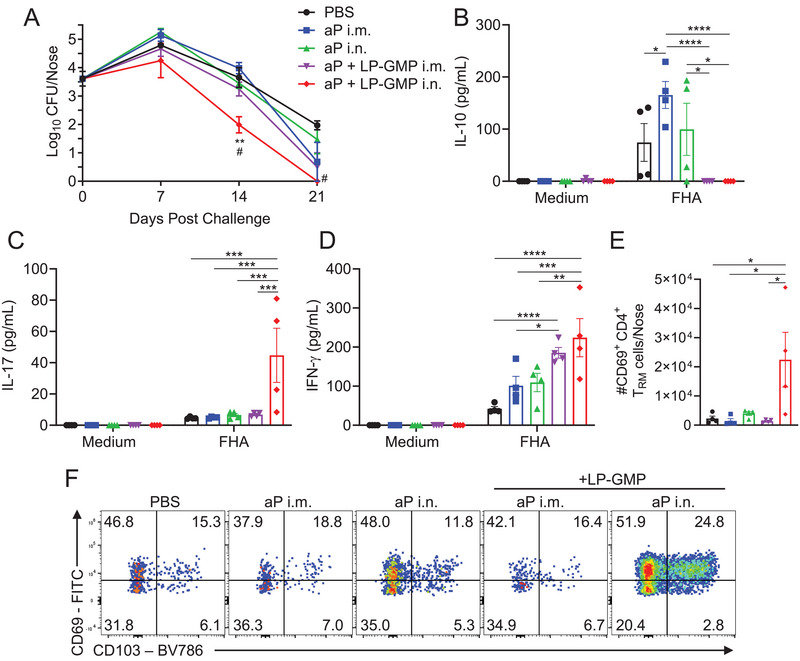
Nasal delivery of aP vaccine formulated with LP‐GMP induces Th17 and Th1‐skewed responses, nasal T_RM_ cells, and confers protection against *B. pertussis* in the nose. Mice were immunized i.m. or i.n. on days 0 and 28 with the aP vaccine (Infanrix; 1/50 human dose), aP + LP‐GMP or PBS as a control. Two weeks following the second immunization, mice were challenged with live *B. pertussis*. (A) CFUs were enumerated in the nasal mucosa 2 h (day 0) and 7‐, 14‐, and 21 days post challenge. Data are mean ±SEM (*n* = 3–4 mice per group, per time‐point), representative of three independent experiments. **p < 0.01 versus aP i.m., #*p* < 0.01 versus aP i.n. by two‐way ANOVA with Tukey's post‐test. (B–D) On the day of challenge, cervical and inguinal LNs and spleens were co‐cultured in triplicate with FHA (0.5 µg/mL) or medium alone for 72 h. Concentrations of IL‐10 (B), IL‐17 (C), and IFN‐γ (D) in supernatants were quantified by ELISA. (E, F) On the day of the challenge, mice were injected i.v. with an αCD45 antibody 10 min prior to euthanasia. Nasal tissue cells were stained with antibodies specific for T_RM_ cell surface markers. Mean absolute numbers of CD69^+^ CD4^+^ T_RM_ cells (E), with representative flow cytometry plots below (**F**). Data shown are mean ± SEM (*n* = 4 mice per group; each symbol represents an individual mouse), and are representative of two (B–D) or three (E, F) independent experiments. *p < 0.05, **p < 0.01, ***p < 0.001, ****p < 0.0001 by two‐way (B–D) or one‐way (E) ANOVA, with Tukey's post‐test.

Immunization with the aP vaccine Infanrix by the i.m. route did not confer protection against nasal infection of mice with *B. pertussis* (Figure 6A). Immunization with the aP vaccine by the i.n. route also failed to alter the course of infection in the nose following *B. pertussis* challenge (Figure 6A). The addition of the mucosal adjuvant with LP‐GMP did not significantly enhance the efficacy of the commercial aP vaccine when delivered by the i.m. route. However, when delivered by the i.n. route, immunization with the aP vaccine and LP‐GMP led to significantly lower bacterial burden in the nasal tissue of mice following aerosol challenge with *B. pertussis* (Figure 6A).

IL‐10 was produced by FHA‐stimulated LN and spleen cells from mice immunized with the aP vaccine by the i.m. or i.n. routes, but not from mice immunized i.n. or i.m. with the aP vaccine formulated with LP‐GMP (Figure 6B). Conversely, antigen‐specific IL‐17 production was detected in LN and spleen cells of mice immunized i.n. with the aP vaccine formulated with LP‐GMP, but not in mice immunized i.m. or i.n. with Infanrix (Figure 6C). IFN‐γ production was also detected in LN and spleen cells from mice immunized i.n. with aP vaccine formulated with LP‐GMP, and this was greater than that induced with the aP vaccine without LP‐GMP (Figure 6D).

FHA‐specific IgG1 and IgG2c were significantly induced in the serum of mice immunized with the aP vaccine with or without LP‐GMP by i.n. or i.m. route. However, the strongest IgG2c response was observed in mice immunized i.m. with the aP vaccine and LP‐GMP, whereas the strongest IgG1 response was induced with the aP vaccine without LP‐GMP delivered by the i.m. route (Figure S9A,B). Immunization with the aP vaccine and LP‐GMP by the i.n. route induced FHA‐specific IgA in the nasal mucosa, whereas immunization with the aP vaccine alone by the i.m. or i.n. route or the aP vaccine and LP‐GMP by the i.m. route failed to induce nasal IgA (Figure S9C).

Finally, CD69^+^ CD4^+^ T_RM_ cells were induced in the nasal tissue of mice immunized i.n. with the aP vaccine and LP‐GMP, but not in mice immunized i.m. or i.n. with aP without LP‐GMP (Figure 6E,F). Furthermore, only the aP vaccine with LP‐GMP delivered by i.n. route induced FHA‐specific IL‐17^+^ or IFN‐γ^+^ CD69^+^CD4^+^ T_RM_ cells in the nasal tissue on the day of challenge (Figure S10). On day 7 post‐*B. pertussis* challenge, the number of IL‐17^+^ CD69^+^ CD4^+^ T_RM_ cells were expanded in mice immunized i.n. with aP and LP‐GMP, but not in mice from any other immunization group (Figure S10).

Together, these data suggest that i.n. route of delivery, when combined with the addition of the strong mucosal adjuvant LP‐GMP, can skew the alum‐adjuvanted aP vaccine toward Th17 and Th1‐dominated responses, promote the induction of nasal CD4^+^ T_RM_ cells and accelerate bacterial clearance from the nasal mucosa following challenge with *B. pertussis*.

## 3 Discussion

The key findings of this study are that commercial alum‐adjuvanted aP vaccines induce IL‐10‐secreting CD4^+^ and CD8^+^ Treg cells. Neutralizing IL‐10 signaling at the time of aP immunization partially reversed aP vaccine‐induced suppression of nasal CD4^+^ T_RM_ responses and enhanced bacterial clearance in the nasal mucosa. While infection with *B. pertussis* and immunization with the wP vaccine leads to robust Th1 and Th17 responses, aP vaccines generate Th2‐skewed T cell responses [8, 9, 11, 34, 44]. IL‐4 production has been linked with suppression of T_RM_ cells in mice immunized with aP vaccines [18]. Our findings demonstrate that in addition to Th2 cells, the aP vaccines also induced Treg cells that express immune checkpoints and secrete the immunosuppressive cytokine IL‐10, which also contributes to suppression of protective T cell responses.

We have previously demonstrated that FHA, a *B. pertussis* antigen in all aP vaccines, promotes IL‐10 production by macrophages and DCs, which suppresses IL‐12 and IFN‐γ production *in vivo* [22]. Human DCs also produce IL‐10 in response to stimulation with FHA [23]. Furthermore, increased IL‐10 production by monocytes following aP vaccination was associated with impaired IFN‐γ responses [45]. A polymorphism in the IL‐10 promoter, linked with higher IL‐10 production, was associated with reduced antigen‐specific T cell proliferation in individuals immunized with the aP vaccine [46]. Moreover, the adjuvant alum also induces IL‐10 production by DCs and macrophages, which suppresses antigen‐specific Th1 responses in mice [25]. Our study demonstrates that peripherally induced Treg cells, which express immune checkpoints and secrete IL‐10, play a role in suppression induced by alum‐adjuvanted aP vaccines.

We have previously reported that IL‐10‐secreting Tr1‐type CD4^+^ Treg cells and IL‐10^+^Foxp3^+^ Treg cells are induced in the lungs of mice infected with *B. pertussis* [21, 47]. In the present study, we found that immunization with the aP vaccine generated CD4^+^ T cells that express Foxp3, PD‐1, and CD49b, with lower expression of LAG‐3. The aP vaccine also induced CD8^+^ T cells that express CD122 and PD‐1, and low levels of Foxp3, and these cells secreted IL‐10. Furthermore, antigen‐specific T cell lines established from mice immunized with the aP vaccine included CD4^+^ T cells that express CD49b, PD‐1, and LAG‐3, and CD8^+^ T cells that express LAG‐3, CD49b, PD‐1, and CD122. Neither CD4^+^ nor CD8^+^ T cells in the antigen‐specific T cell line express Foxp3, which is consistent with the expansion of peripheral Treg cells that normally do not express Foxp3. These *B. pertussis*‐specific CD4^+^ and CD8^+^ Treg cell lines produced IL‐10, IFN‐γ, and IL‐5, but little or no IL‐17, and instead suppressed IL‐17 production by Th17 cells from convalescent mice. CD122^+^CD8^+^ Treg cells that express PD‐1 have previously been shown to suppress CD4^+^CD25^−^ effector T cell proliferation through IL‐10 production [41]. Furthermore, in an autoimmune disease model, CD122^+^CD8^+^ Treg cells prevented the development of disease through IL‐10‐mediated suppression of IL‐17 production [48]. The current study reveals, for the first time, that CD8^+^ Treg cells can also be induced by immunization with protein vaccines formulated with alum as the adjuvant. Our findings demonstrate that immunization with licensed pertussis vaccines induces antigen‐specific CD8^+^ Treg cells that secrete IL‐10, which, together with CD4^+^ Treg cells, suppress activation of IL‐17‐secreting T cells, known to play a crucial role in protective adaptive immunity to *B. pertussis*.

Our demonstration that vaccine‐induced IL‐10‐secreting Treg cells inhibit the development and/or activation of Th17 cells is consistent with studies on *S. aureus*. IL‐10 induction by a *S. aureus* vaccine inhibited IL‐17 responses, impaired protection against *S. aureus* systemic challenge, and was reversed by neutralization of IL‐10 at the time of challenge [49]. Furthermore, blockade of IL‐10 signaling in mice immunized with *S. aureus* clumping factor A antigen vaccines, adjuvanted with CpG or LP‐GMP, increased IL‐17 and IL‐22‐secreting T cells in the skin and enhanced protective efficacy against subcutaneous *S. aureus* challenge [31]. It has also been demonstrated that genetic or antibody neutralization of IL‐10 enhanced protection against *M. tuberculosis* infection of the lung induced by immunization with BCG, and this was associated with enhanced Th1 or Th17 responses [29, 30].

Our study has demonstrated that blocking IL‐10 signaling enhances the efficacy of an alum‐adjuvanted vaccine by reversing the suppression of T_RM_ cells. The beneficial effect of IL‐10 blockade was observed when mice were treated with the anti‐IL‐10R antibody at the time of immunization with the aP vaccine, but also when IL‐10 was blocked during *B. pertussis* challenge. This suggests that IL‐10 induced with the aP vaccine may attenuate the induction of protective T cells but may also suppress the expansion and/or activation of T_RM_ cells in the respiratory tissue. Our data provide further evidence that the aP vaccines do not prevent nasal infection because they suppress the induction or recruitment of IL‐17‐secreting CD4^+^ T_RM_ cells to the respiratory tissue and suggest that this is in part mediated by vaccine‐induced IL‐10‐secreting Treg cells. It has previously been shown that IL‐10 produced by CD4^+^ Treg cells was necessary for the maturation of protective memory CD8^+^ T cells in mice infected with lymphocytic choriomeningitis virus [50]. However, CD8^+^ T cells enhance rather than protect against infection with *B. pertussis* [11]. Our current study shows that IL‐10‐secreting CD8^+^ T cells, together with CD4^+^ Tregs cells, induced with an aP vaccine, suppress protective CD4^+^ T_RM_ cells, which is consistent with this finding.

The translation of our findings to the vaccination of humans will not be straightforward. Even transient blocking of IL‐10 signaling during immunization is unlikely to be a practical means of enhancing vaccine efficacy in infants. An alternative approach may be to add a more powerful adjuvant to the current alum‐adjuvanted pertussis vaccine. Alum promotes Th2 responses but does not induce strong Th1 or Th17 responses, which require the T cell polarizing cytokines IL‐12 and IL‐23. Instead, it induces IL‐10 and inhibits IL‐12 production by DCs [25, 51, 52]. Conversely, adjuvants, such as oil‐in‐water emulsions or TLR agonists that activate innate immune cells, which direct the induction of potent cellular immune responses, have already been licensed for use in humans [53]. We have previously reported that LP‐GMP induces IL‐12 and IL‐23 production by DCs, and when used as an adjuvant for an experimental aP vaccine delivered by the nasal route, it promoted the induction of IL‐17^+^ CD4^+^ T_RM_ cells and conferred long‐term protective immunity against *B. pertussis* in the nose [38]. In the present study, we demonstrate that the addition of LP‐GMP to the commercial aP vaccine, administered by the i.n. route, led to a significant reduction in bacterial burden in the nasal mucosa following *B. pertussis* challenge. This was associated with reduced IL‐10‐secreting T cells and enhanced antigen‐specific IL‐17‐secreting T cell responses. These findings underline the potential for the development of a more effective pertussis vaccine using potent mucosal adjuvants that promote, rather than suppress, T_RM_ cell responses at the site of infection in the nasal mucosa.

### 3.1 Data Limitations and Perspectives

Current aP vaccines do not prevent nasal infection with *B. pertussis* or sustain immunity against pertussis disease, but instead suppress the induction of protective respiratory T_RM_ cells. Our study has addressed the possible mechanisms involved and demonstrated that immunization of mice with aP vaccines generates CD4^+^ and CD8^+^ Treg cells that express markers of thymically derived Treg cells and antigen‐specific peripherally induced Treg cells. These Treg cells suppress IL‐17 production by *B. pertussis*‐specific effector T cells, thereby limiting their ability to confer protective immunity in the nasal mucosa. Our results suggest that the suppression of T_RM_ cells involves IL‐10, but we have not addressed the possible co‐operative role of Th2 cytokines, especially IL‐4. While our findings provide a plausible explanation for the shortcomings of aP vaccines in humans, a limitation of the study is that it only involved experiments in a mouse model. Further studies will be required to confirm these findings in humans.

## 4 Materials and Methods

### 4.1 Mice

Female C57BL/6 and CD45.1 mice were bred in‐house from established colonies and housed in a specific pathogen‐free facility in the Comparatives Medicines Unit, Trinity College Dublin. Unless otherwise stated all experiments were carried out with C57BL/6 mice. Mice were 6–12 weeks old at the initiation of experiments. Mice were euthanized by asphyxiation with CO_2_. Animal experiments were carried out with the approval of the TCD Animal Research Ethics Committee and conducted in accordance with European Union regulations under licence (AE19126/P042) from the Irish Health Products Regulatory Authority.

### 4.2 Immunization and Vaccines

In prime‐boost studies, mice were immunized on days 0 and 28, by i.p. (200 µL/mouse), i.m. (50 µL/mouse) or i.n. route (25 µL/mouse) with 1/20th or 1/50th of the human dose of the primary hexavalent vaccine containing aP, Infanrix (GlaxoSmithKline), or 1/10^th^ of the human dose of the booster TdaP vaccine, Boostrix (GlaxoSmithKline). The adjuvant LP‐GMP was formulated with cyclic‐di‐GMP (10 µg/mouse; Invivogen) and LP1569 (50 µg/mouse, EMC Microcollections) and was combined with Infanrix (1/50 human dose). For i.n. immunization, mice were anesthetized with ketamine (50 µg/g; Chanelle Pharmaceuticals) and xylazine (10 µg/g; Chanelle Pharmaceuticals), and anesthesia was reversed with atipamezole (20 µg/mouse; Animalcare). Tissues were collected on day 3 or at 1 or 2 weeks post boost. Mice were challenged with *B. pertussis* at 2 weeks post boost.

### 4.3 Detection of Tissue‐Resident Immune Cells

Mice were injected intravenously (i.v.) with 200 µL PE‐conjugated anti‐CD45 (αCD45) antibody (1.5 µg/mouse) in sterile PBS, 10 min prior to euthanasia. This allows for the discrimination of antibody‐labeled circulating immune cells (CD45i.v.^+^) from tissue‐resident immune cells (CD45i.v.^−^), which are not labeled by the antibody, by flow cytometry, using a validated method [17].

### 4.4 Treatment of Mice with Antibodies *in Vivo*

To neutralize IL‐10 signaling *in vivo*, mice were injected i.p. with 200 µL of anti‐IL‐10 receptor alpha antibody (αIL‐10R; 200 µg/mouse; BioXCell) or IgG1 isotype control antibody (rat IgG1 isotype control, anti‐horseradish peroxidase (HRP); 200 µg/mouse; BioXCell) 1 day prior to and 3 days following aP prime and boost, or mice were injected on 1 day and 4 h prior to *B. pertussis* challenge and on days 4, 8, 12, and 16 after challenge.

### 4.5 Aerosol Challenge with Live *B. pertussis*

Mice were aerosol challenged with live *B. pertussis* (Bp338 strain, 1 × 10^9^ CFU/mL), administered via a nebulizer (PARI TurboBOY SX or PARI LC Sprint Nebuliser) for 10 min. CFU counts were enumerated by culturing nasal tissue digests on Bordet Gengou agar.

### 4.6 Isolation of Nasal, Lung, Spleen, and LN Cells

Lung and nasal tissue were digested as described [19]. LNs, spleens, digested lung, and nasal tissue were mashed through a 70 µm (lung, nasal tissue, spleen) or 40 µm strainer (LNs). Tissues were collected and processed in complete RPMI medium or in sterile PBS for the nasal tissue to allow the use of nasal digest for the detection of bacteria. Immune cells from the lung were purified by density centrifugation by resuspending lung cells in 7 mL of 30% isotonic Percoll (GE Healthcare) and centrifuging cells at 1500 rpm for 20 min at room temperature, with the brake turned off. The upper mucus layer and remaining liquid were carefully removed and discarded, and the cell pellet, containing purified lung immune cells, was used for *ex vivo* cell culture.

### 4.7 Generation of *B. pertussis*‐Specific T Cell Lines

Inguinal and popliteal LNs were collected 7 days after booster immunization with an aP vaccine (Boostrix; 1/10 human dose). FHA‐ and *B. pertussis*‐specific T cell lines were established by stimulating LN and spleen cells (2 × 10^6^ cells/mL), at a 6:4 LN:spleen ratio, with FHA (2 µg/mL) or sBp (5 µg/mL) in the presence of IL‐2 (10 ng/mL), with or without IL‐15 (5 ng/mL), in cRPMI, supplemented with 1X Insulin Transferrin Selenium (ITS; Gibco). On day 8, cells were harvested and T cell lines (2 × 10^5^ cells/mL) were co‐cultured with irradiated naive spleen cells, from CD45.1 mice, as APCs (2 × 10^6^ cells/mL), and FHA (2 µg/mL) or sBp (5 µg/mL) in the presence of IL‐2 (10 ng/mL) with or without IL‐15 (5 ng/mL) at 37°C in 5% CO_2_. On day 16, this process was repeated. Cells were fed with IL‐2 (10 ng/mL) with or without IL‐15 (5 ng/mL) on days 4, 12, and 20. On days 8 and 16, a proportion of the T cell lines were cultured *ex vivo* and were analyzed by flow cytometry.

### 4.8 T Cell Suppression Assay

CD4^+^ T_eff_ cells were isolated from LNs (cervical, axillary, brachial) and lungs of *B. pertussis* convalescent mice, using the CD4^+^ EasySep Isolation Kit as per the manufacturer's protocol (StemCell Technologies), and cells were pooled at a 3:7 LN:lung ratio. Convalescent CD4^+^ T_eff_ cells (0.5 × 10^5^ cells/well) were co‐cultured with Treg cell lines (0.5 × 10^5^, 2.5 × 10^5^, 5 × 10^5^ cells/well) and irradiated splenic APCs from naïve CD45.1 mice (5 × 10^5^ cells/well) and stimulated with FHA (2 µg/mL) in 96‐well U‐bottomed cell culture plates, at 37°C in 5% CO_2_. After 4 days, cell supernatants were harvested, and IL‐17 and IL‐10 concentrations were quantified by ELISA using DuoSet ELISA kits (R&D Systems) according to the manufacturer's protocol.

### 4.9 *Ex Vivo* Stimulation of Spleen, LN, PEC, Lung Immune Cells, and T Cell Lines

PECs (2 × 10^5^ cells/well), lung lymphocytes (1 × 10^5^ cells/well) and irradiated naïve APCs (4 × 10^5^ cells/well), spleen and LN cells (1:1 ratio, 4 × 10^5^ cells/well) or LN cells alone (2 × 10^5^ cells/well) were cultured in cRPMI in the presence of *B. pertussis* antigens, PRN (1 µg/mL; List Laboratories), FHA (0.5 or 2 µg/mL; The Native Antigen Company), sBp (5 µg/mL; laboratory‐made) or polyclonal T cell stimuli PMA (25 ng/mL) and an anti‐CD3ε antibody (1 µg/mL; αCD3), or with medium only as a negative control. T cell lines (0.4 × 10^5^ cells/well) were cultured with irradiated APCs from CD45.1 mice (4 × 10^5^ cells/well), with FHA (2 µg/mL) for FHA‐specific T cell lines, or with sBp (5 µg/mL) for *B. pertussis* T cell lines, or with ITS‐supplemented cRPMI as a negative control. Cells were cultured in U‐bottomed (or F‐bottomed for PEC) 96‐well cell culture plates for 72 h at 37°C, in 5% CO_2_, and cell supernatants were analyzed by ELISA.

### 4.10 Detection of Cytokines by ELISA

IL‐17 and IL‐10 concentrations were quantified using DuoSet ELISA kits (R&D Systems), and IL‐5 and IFN‐γ concentrations were assessed using BD Pharmingen ELISA antibodies according to manufacturers’ protocols and were read using a VersaMax microplate reader (Molecular Devices).

### 4.11 Flow Cytometric Analysis of Nasal Tissue, PECs, and T Cell Lines

Red blood cells in nasal tissue samples were lysed using ammonium chloride buffer. PECs, T cell lines, and nasal cells were stained with LIVE/DEAD Aqua (1:600, Invitrogen), extracellular antibodies, and fixed with 2% paraformaldehyde (PFA; ThermoFischer Scientific), if not performing intracellular staining. To examine cytokine production by nasal T_RM_ cells, nasal immune cells were stimulated with PMA (50 ng/mL; Sigma‐Aldrich) and ionomycin (500 ng/mL; Sigma‐Aldrich) in the presence of brefeldin A (5 µg/mL; Sigma‐Aldrich) for 4 h at 37^°^C, in 5% CO_2_. To examine FHA‐specific cytokine production, nasal cells were stimulated with FHA (2 µg/mL) and antibodies specific for co‐stimulatory molecules CD49d (1 µg/mL) and CD28 (1 µg/mL) for 20 h, with brefeldin A (5 µg/mL) and monensin (1 µg/mL) added for the final 4 h. For intracellular staining, cells were permeabilized and fixed using the Foxp3/transcription factor staining buffer kit (ThermoFischer Scientific), according to the manufacturer's protocol. Flow cytometric data were acquired with an Aurora (CytekBio) full‐spectrum analyzer using SpectroFlo (CytekBio) software. Data were analyzed using FlowJo 10 (Treestar) software. Details of gating strategies are shown in Figures S11 and S12, and details of antibodies used in the study are shown in Table S1.

### 4.12 Detection of FHA‐specific Antibodies by ELISA

Serum and nasal tissue were collected 2 weeks post immunization, and antibody responses specific for the *B. pertussis* antigen FHA were quantified by ELISA. Samples were added to medium‐bind ELISA plates coated with FHA (1 µg/mL). Bound antibodies were detected using HRP‐conjugated IgG1 (Invitrogen), IgG2c (Southern Biotech), and IgA (Southern Biotech). Antibody concentrations are expressed as Log_10_ mean endpoint titers (±SEM), determined by extrapolation of the linear part of the titration curve to the baseline value obtained from samples taken from nonimmunized mice.

### 4.13 Immunocytochemistry, Confocal Imaging, and Imaris Reconstruction

Spleen cells or spleen cells and mediastinal LN cells (1:1 ratio) from immunized and control mice were cultured at 8 × 10^5^ cells/well for 20 h with FHA (2 µg/mL) and anti‐CD49d and anti‐CD28 (both at 1 µg/mL), with brefeldin A (5 µg/mL) and monensin (1 µg/mL) added for the final 4 h. Cells were fixed with 4% PFA, and blocked using 5% fetal bovine serum in PBS. Surface staining was performed using an anti‐mouse Alexa Fluor 647‐conjugated CD8 antibody (Clone: 53–6.7, Biolegend). Cells were then permeabilized using 0.3% Triton X‐100 in and intracellular staining was performed using anti‐mouse CoraLite Plus 488‐conjugated IL‐10 antibody (Proteintech). Cells were mounted on microscopy slides using the Cytospin3 (Shandon) and counterstained with mounting media containing DAPI (Ibidi). Raw images were acquired using a Leica SP8 confocal microscope (x40 objective). 10–20 z‐stack planes were taken with 0.3 µm spacing, and images were processed on the LAS X Life Science Microscope Software Platform. For visualization of IL‐10 expression in CD8^+^ T cells, after background subtraction using median and Gaussian filters, CD8^+^ T cells and IL‐10 were 3D surface‐rendered with 0.2 and 0.001 µm smoothing, respectively, using Imaris 10.2 (Bitplane) software. A mask was then applied within CD8^+^ reconstructed T cells for IL‐10 to visualize IL‐10‐producing CD8^+^ T cells.

### 4.14 Statistical Analysis

Statistical analysis was carried out using GraphPad Prism 10. Paired Student's *t*‐test was used to analyze differences in cytokine production from Treg cell lines. Otherwise, unpaired student's *t*‐test or Mann‐Whitney *U* test was used to analyze statistical significance between two groups. One or two‐way analysis of variance (ANOVA) with Šidák's, Tukey's, or Dunnett's post‐test was used to analyze statistical significance between three or more groups. Error bars indicate mean ± standard error of the mean (SEM) or standard deviation (SD), as indicated in figure legends. *P*‐values of 0.05 or lower were considered to be statistically significant.

## Author Contributions

Kingston H. G. Mills supervised the project. Kingston H. G. Mills and Mieszko M. Wilk conceived the study and interpreted the data. Caitlín Ní Chasaide executed the experiments and analyzed and interpreted the data. Pauline Schmitt, Béré K. Diallo, Lisa Borkner, Charlotte M. Leane, Seyed Davoud Jazayeri, Sreeram Udayan, Eoin O'Neill, and Lucy M. Curham assisted with the acquisition of the data. Barry Moran assisted with the flow cytometry studies. Caitlín Ní Chasaide and Kingston H. G. Mills wrote and revised the manuscript. All other authors provided critical feedback on the manuscript.

## Ethics Statement

All animal experiments were approved by the Trinity College Dublin Animal Research Ethics Committee.

## Conflicts of Interest

Kingston Mills is the co‐founder of a Start‐up company and has collaborative research funding from and acts as a consultant to Pharmaceutical and Biotech companies. Kingston Mills, Béré Diallo, Caitlín Ní Chasaide, and Pauline Schmitt are inventors on a patent application around a novel vaccine adjuvant.

## Peer Review

The peer review history for this article is available at https://publons.com/publon/10.1002/eji.202451630.

## Supporting information




**Supporting File 1**: eji6017‐sup‐0001‐SuppMat.pdf.

## Data Availability

The data sets generated during the current study are available from the corresponding author upon reasonable request.
