# Supplementary material for: Acellular Pertussis Vaccines Induce CD8+ and CD4+ Regulatory T Cells That Suppress Protective Tissue‐Resident Memory CD4+ T Cells, in Part via IL‐10
Source: Eur J Immunol. 2025 Jul 9;55(7):e51630. doi: 10.1002/eji.202451630 (PMC12238839; doi:10.1002/eji.202451630)
Supplement: Supplementary file 1 — Supporting File 1: eji6017‐sup‐0001‐SuppMat.pdf. [file EJI-55-e51630-s001.pdf]

# Supplementary Figures and Table

**Acellular pertussis vaccines induce CD8⁺ and CD4⁺ regulatory T cells**

**that suppress protective tissue-resident memory CD4⁺ T cells, in part via IL-10**

Caitlín Ní Chasaide, Pauline Schmitt, Béré K. Diallo, Lisa Borkner, Charlotte M. Leane, Seyed Davoud Jazayeri, Sreeram Udayan, Eoin O'Neill, Lucy M. Curham, Barry Moran, Mieszko M. Wilk and Kingston H.G. Mills

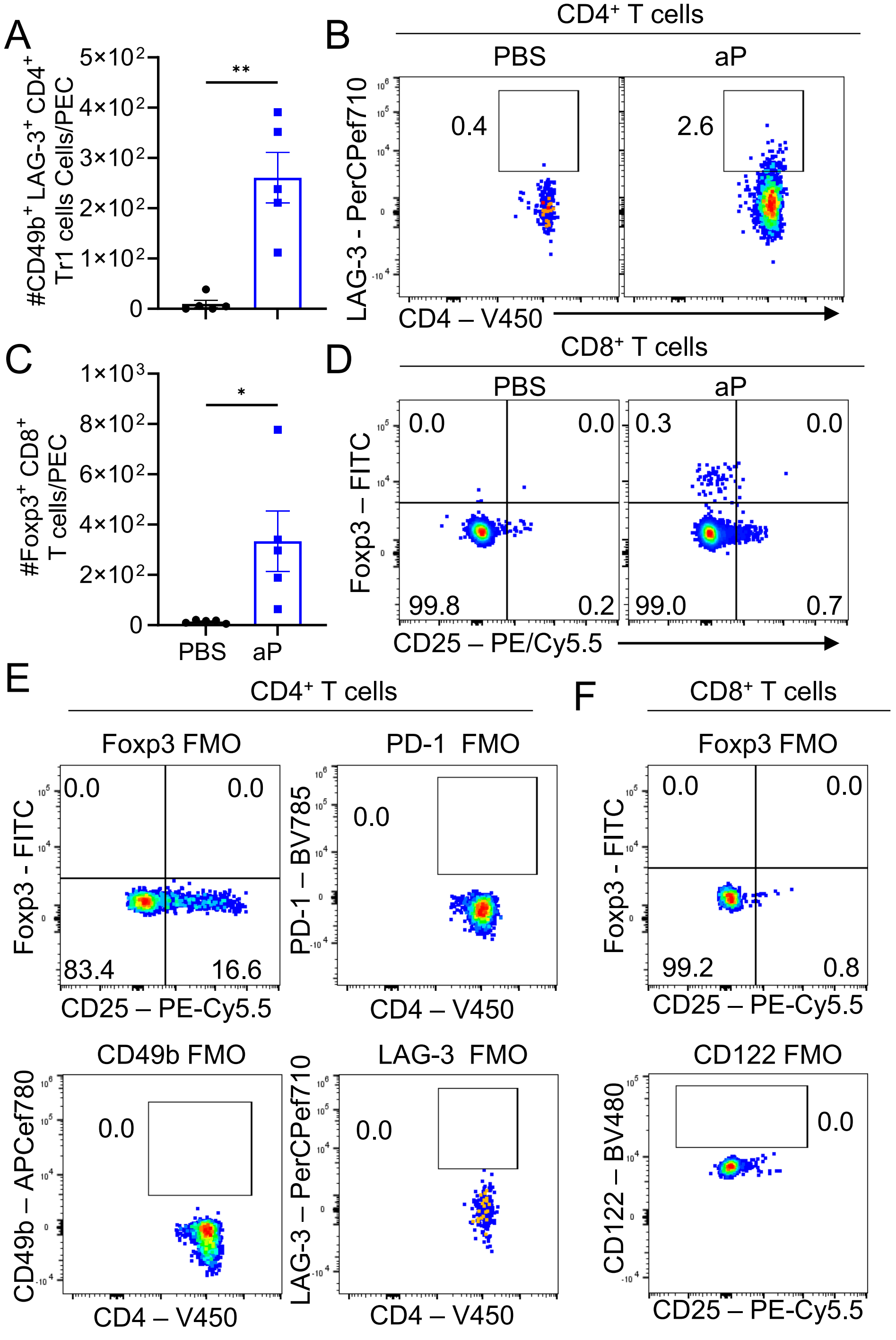


**Figure S1**. **The commercial aP vaccine induces CD4$^{+}$ and CD8$^{+}$ T cells, which express regulatory markers, at the site of immunization.** Mice were immunized i.p. with the aP vaccine (Infanrix; 1/50 human dose) on day 0 and 28, or with PBS as control. 7 days after second immunization, PECs were harvested and stained with cell surface markers CD11b, CD19, CD3, CD4, CD8, CD49b, LAG-3 and intranuclearly for Foxp3. Mean absolute numbers (**A**) and representative flow cytometry plots (**B**) of LAG-3$^{+}$CD49b$^{+}$CD4$^{+}$CD19$^{-}$CD11b$^{-}$ T cells. Mean absolute numbers (**C**) and representative flow cytometry plots (**D**) of Foxp3$^{+}$CD8$^{+}$CD19$^{-}$CD11b$^{-}$ cells. Data shown are mean ±SEM (n=5 mice per group), from one experiment. *$p<0.05$, **$p<0.01$ by Mann-Whitney test. Fluorescence minus one (FMO) controls for CD4$^{+}$ (**E**) and CD8$^{+}$ (**F**) T cell data shown in Fig. 1 and Fig. S1.

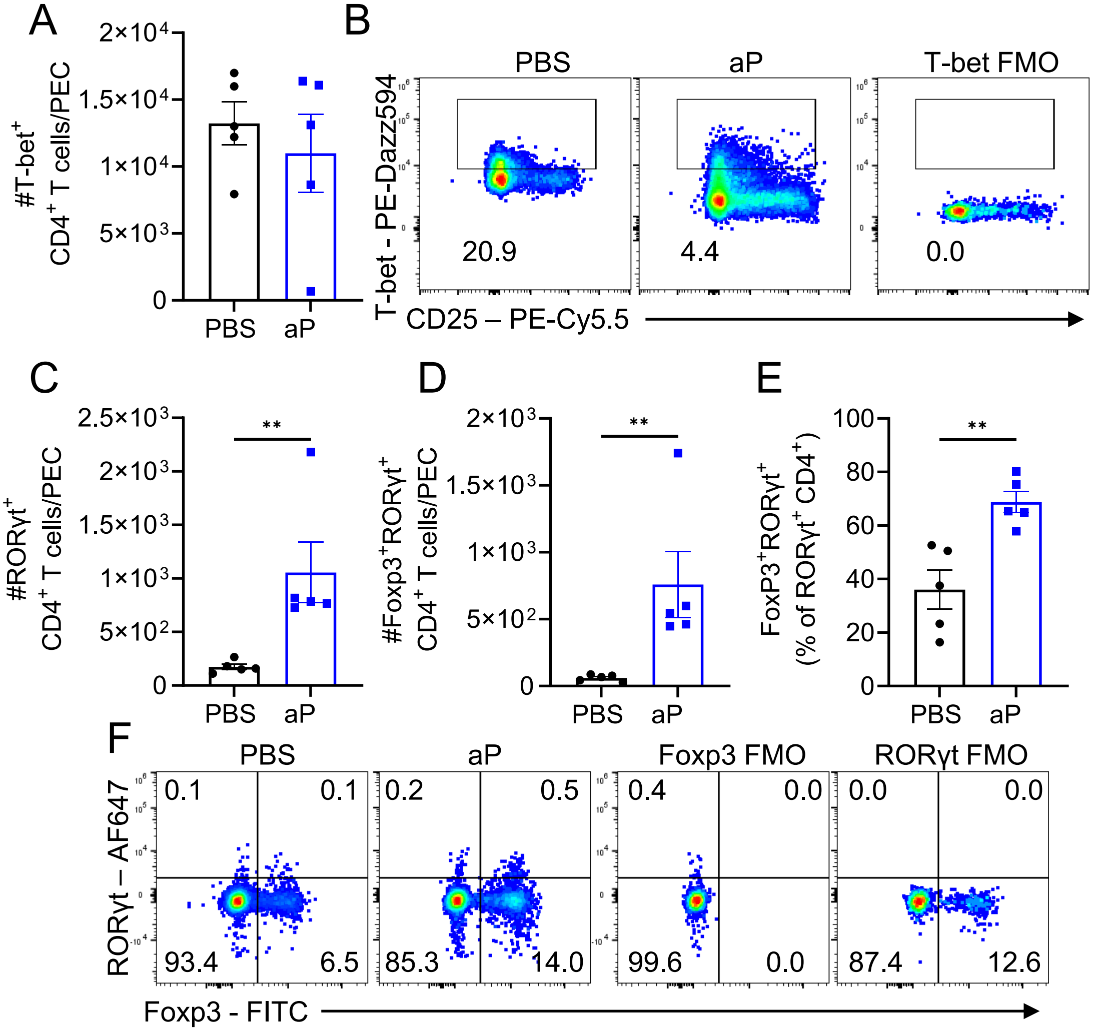


**Figure S2**. **The commercial aP vaccine induces RORγt**$^{+}$**Foxp3**$^{+}$ **but not T-bet**$^{+}$ **CD4**$^{+}$ **T cells** Mice were immunized i.p. with the aP vaccine (Infanrix; 1/50 human dose) on day 0 and 28, or with PBS as control. 7 days after second immunization, PECs were harvested and stained with cell surface markers CD11b, CD19, CD3, CD4, and intranuclearly for Foxp3, T-bet and RORγt. Mean absolute numbers (**A**) and representative flow cytometry plots (**B**) of T-bet$^{+}$CD4$^{+}$CD19$^{-}$CD11b$^{-}$ cells. Mean absolute numbers of RORγt$^{+}$ (**C**) and Foxp3$^{+}$RORγt$^{+}$ (**D**) CD4$^{+}$CD19$^{-}$CD11b$^{-}$ cells, mean frequencies of Foxp3$^{+}$RORγt$^{+}$ cells in total RORγt$^{+}$CD4$^{+}$ cells (**E**), and representative flow cytometry plots (**F**). Data shown are mean ±SEM (n=5 mice per group), from one experiment. **$p<0.01$ by Mann-Whitney test.

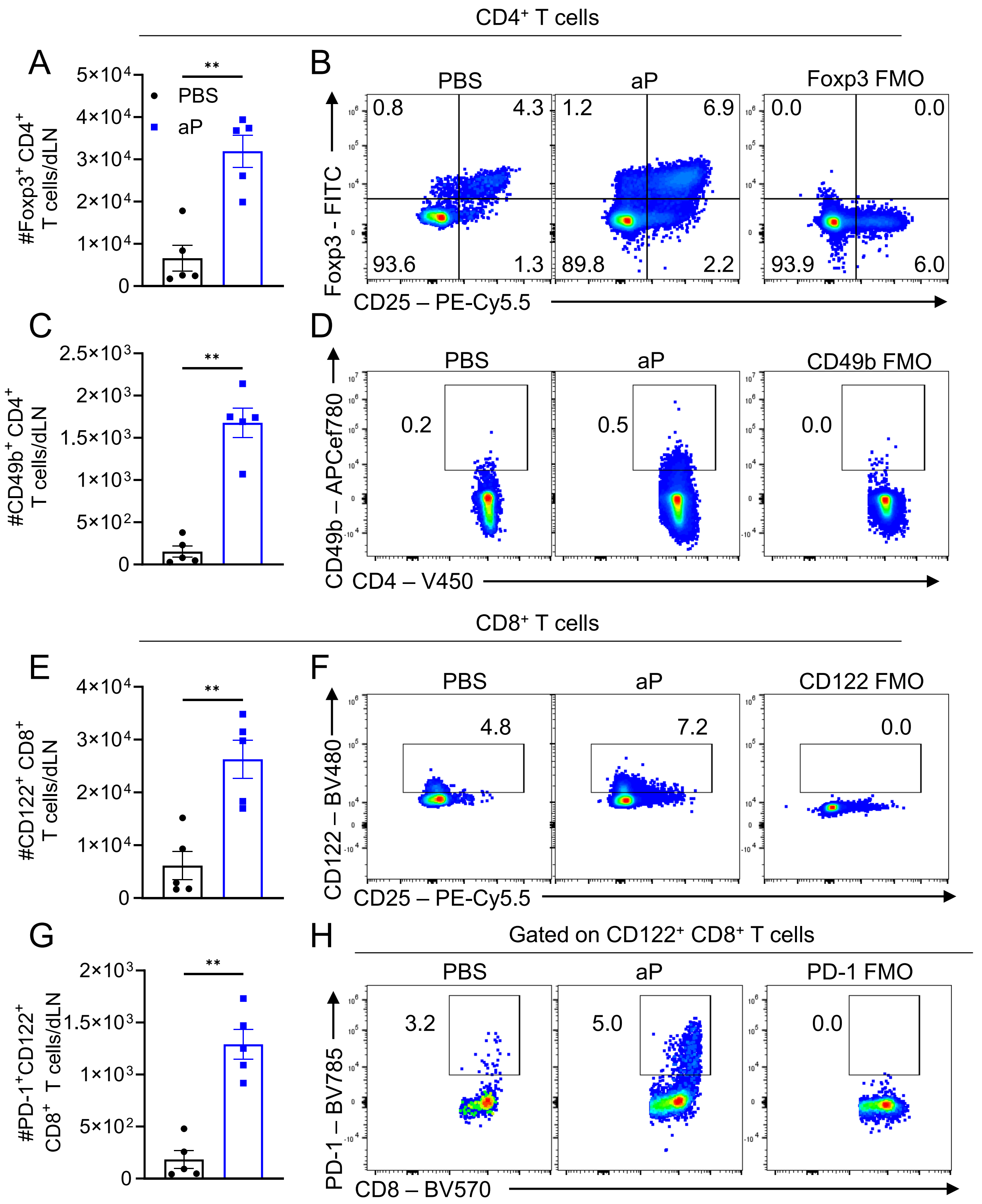


**Figure S3**. **The commercial aP vaccine induces CD4$^+$ and CD8$^+$ T cells, which express regulatory markers, in draining mediastinal LNs.** Mice were immunized i.p. with the aP vaccine (Infanrix; 1/50 human dose) on day 0 and 28, or with PBS as control. 7 days after second immunization, mediastinal LNs (dLNs) were harvested and stained with cell surface markers CD11b, CD19, CD3, CD4, CD8, CD49b, CD122, PD-1 and intranuclearly for Foxp3. Mean absolute numbers (**A**) and representative flow cytometry plots (**B**) Foxp3$^+$CD4$^+$CD19$^-$CD11b$^-$ cells. Mean absolute numbers (**C**) and representative flow cytometry plots (**D**) of CD49b$^+$CD4$^+$CD19$^-$CD11b$^-$ cells. Mean absolute numbers (**E**) and representative flow cytometry plots (**F**) of CD122$^+$CD8$^+$CD19$^-$CD11b$^-$ cells. Mean absolute numbers (**G**) and representative flow cytometry plots (**H**) of PD-1$^+$CD122$^+$CD8$^+$CD19$^-$CD11b$^-$ cells. Data shown are mean ±SEM (n=5 mice per group), from one experiment. **p<0.01 by Mann-Whitney test.

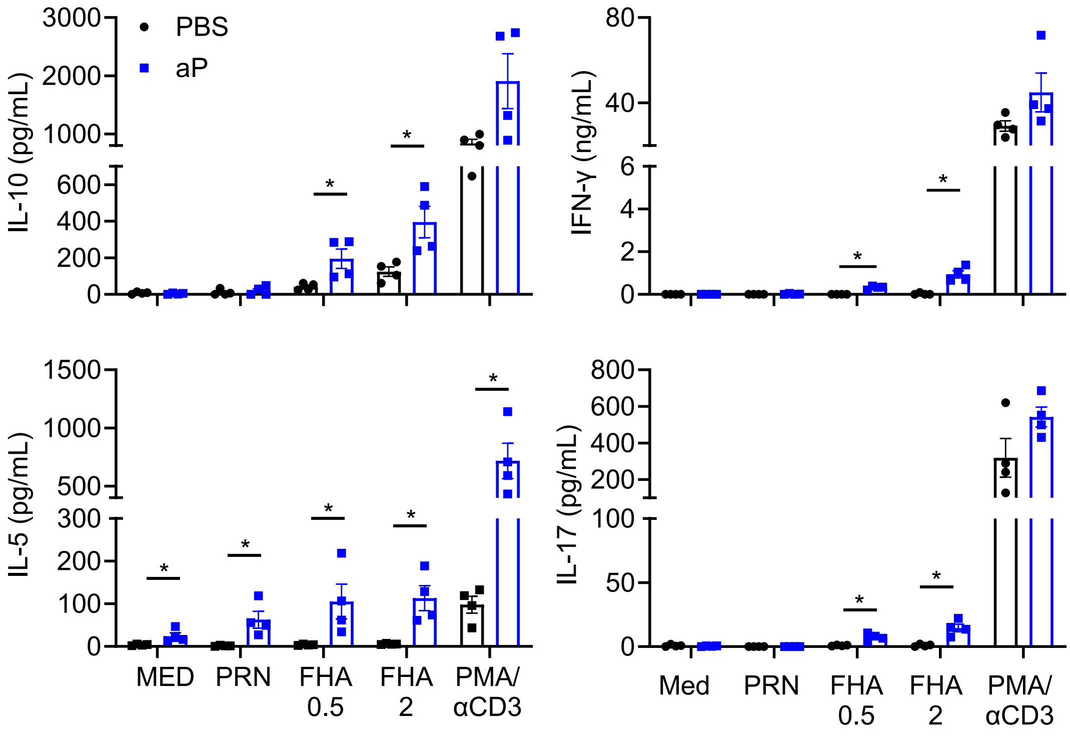


**Figure S4. Immunization of mice with Infanrix induces FHA-specific IL-10.** Mice were immunized i.m. with Infanrix (1/20 human dose) or with PBS as control on day 0 and 28. On day 35, inguinal and popliteal dLNs and spleens were collected and co-cultured with PRN (1 μg/mL), FHA (2 μg/mL), PMA (25 ng/mL) and anti-CD3 (1 μg/mL; αCD3), or medium alone for 72 h. Concentrations of IL-10, IFN-γ, IL-5 and IL-17 in supernatants were quantified by ELISA. Data shown are mean ±SEM (n=4 per group) with each symbol representing triplicate culture for an individual mouse (For PMA/αCD3 stimulation condition, data shown are mean ±SEM (n=4 per group) with each symbol representing single well culture for an individual mouse). *$p<0.05$ by Mann-Whitney test.

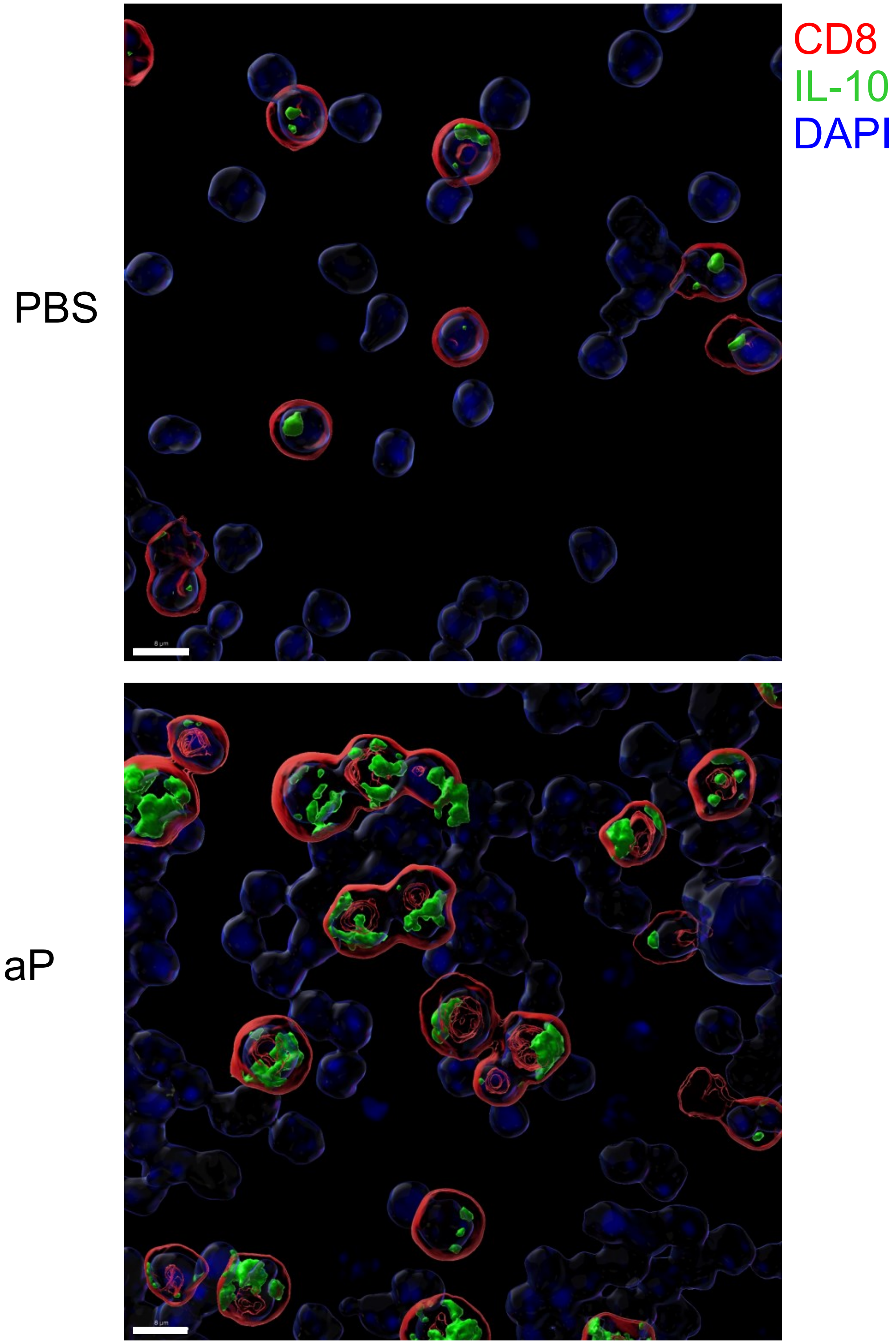


**Figure S5. Intramuscular immunization with commercial aP vaccine induces FHA-specific IL-10-producing CD8$^{+}$ T cells in the spleen.** Mice were immunized i.m. with aP vaccine (Boostrix; 1/10 human dose) or with PBS as control on day 0 and 28. On day 35, spleens were harvested. Representative Imaris reconstruction of IL-10$^{+}$ CD8$^{+}$ T cells in spleen, following 20 h culture of spleen cells (8x10$^{5}$ cells/well) with FHA (2 µg/mL) anti-CD49d and anti-CD28 (both at 1 µg/mL), with brefeldin A (5 µg/mL) and monensin (1 µg/mL) added for final 4 h, prior to immunocytochemistry staining. Representative image from 4 mice per group in one experiment. Scale bars, 8 µm.

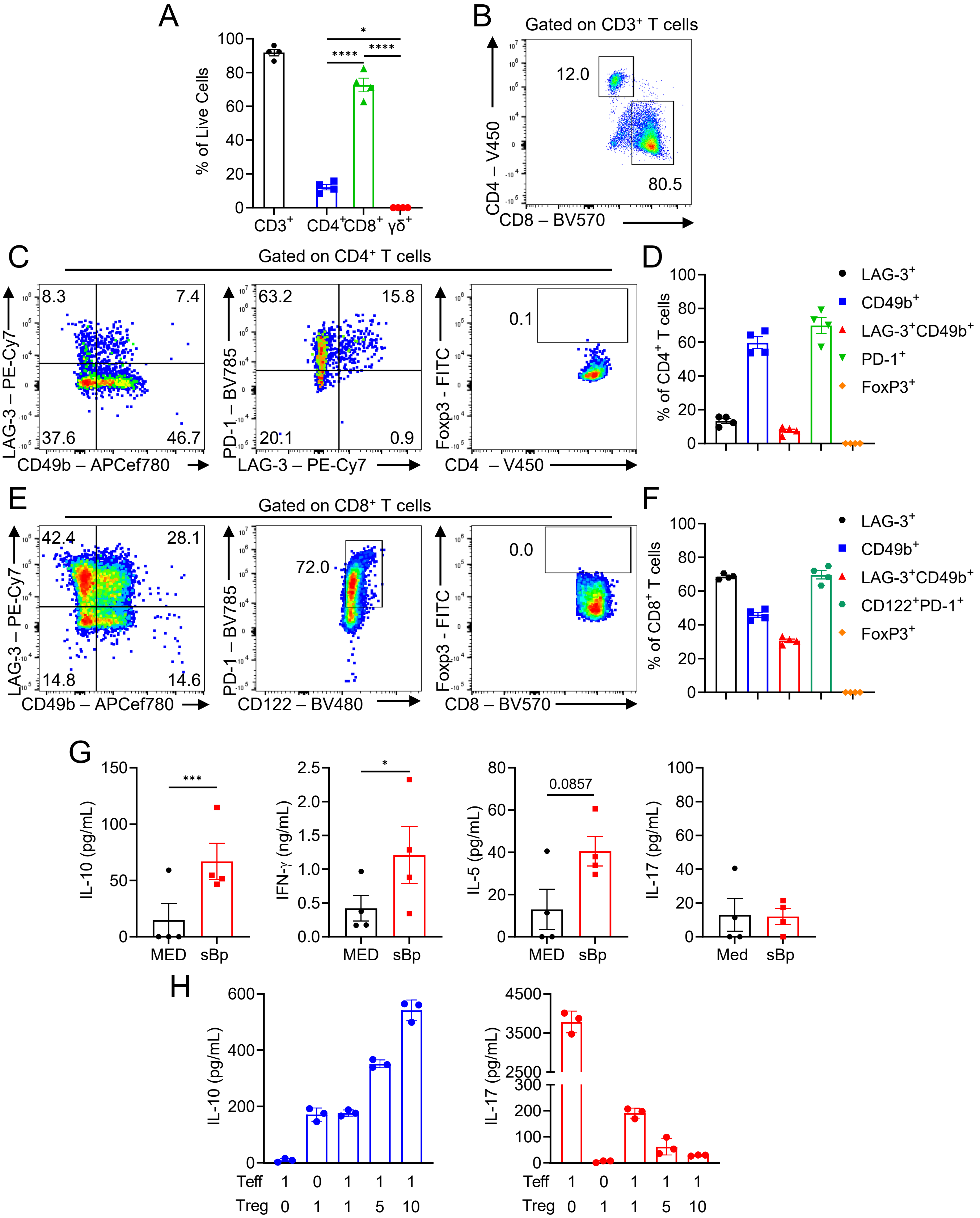

A
% of Live Cells
CD3+
CD4+
CD8+
γδ+
B
Gated on CD3+ T cells
CD4 – V450
CD8 – BV570
12.0
80.5
C
Gated on CD4+ T cells
LAG-3 – PE-Cy7
CD49b – APCef780
PD-1 – BV785
LAG-3 – PE-Cy7
Foxp3 - FITC
CD4 – V450
D
% of CD4+ T cells
LAG-3+
CD49b+
LAG-3+CD49b+
PD-1+
FoxP3+
E
Gated on CD8+ T cells
LAG-3 – PE-Cy7
CD49b – APCef780
PD-1 – BV785
CD122 – BV480
Foxp3 - FITC
CD8 – BV570
F
% of CD8+ T cells
LAG-3+
CD49b+
LAG-3+CD49b+
CD122+PD-1+
FoxP3+
G
IL-10 (pg/mL)
IFN-γ (ng/mL)
IL-5 (pg/mL)
IL-17 (pg/mL)
MED
sBp
Med
0.0857
H
IL-10 (pg/mL)
IL-17 (pg/mL)
Teff
Treg

**Figure S6. *B. pertussis*-specific T cell lines established from aP-immunized mice produce antigen-specific IL-10, IL-5 and IFN-γ and suppress antigen-specific Th17 cells.** Mice were immunized i.m. with aP vaccine (Boostrix; 1/10 human dose) on day 0 and 28. On day 35, T cell lines were generated by culturing cells with sBp in the presence of IL-2 and IL-15. T cells were stained with antibodies specific for T cell surface markers and analysed by flow cytometry. Frequencies of live $CD3^{+}$ $CD4^{+}$, $CD8^{+}$ and $\gamma\delta^{+}$ T cells (**A**) and representative flow cytometry plots for CD4 versus CD8 (pre-gated on $CD3^{+}CD19^{-}CD11b^{-}CD45.2^{+}$ cells) (**B**). Representative flow cytometry plots **(C)** and mean frequencies **(D)** of LAG-$3^{+}$, $CD49b^{+}$, LAG-$3^{+}CD49b^{+}$, PD-$1^{+}$ and $Foxp3^{+}$ $CD4^{+}$ T cells (all pre-gated on $CD4^{+}CD19^{-}CD11b^{-}CD45.2^{+}$ cells). Representative flow cytometry plots **(E)** and mean frequencies **(F)** of LAG-$3^{+}$, $CD49b^{+}$, LAG-$3^{+}CD49b^{+}$, $CD122^{+}$PD-$1^{+}$ and $Foxp3^{+}$ $CD8^{+}$ T cells (all pre-gated on $CD8^{+}CD19^{-}CD11b^{-}CD45.2^{+}$ cells). (**G**) Cells from a *B. pertussis*-specific T cell lines were stimulated with sBp or medium only in the presence of splenic irradiated APCs for 72 h. Concentrations of IL-10, IL-5, IFN-γ and IL-17 were quantified by ELISA. Data shown are ±SEM (n=4 per group), with each symbol representing T cell line established from an individual mouse. *$p<0.05$, ****$p<0.0001$ by one-way ANOVA with Tukey's post-test (**A**) or *$p<0.05$, ***$p<0.001$ by paired t-test (**G**). (**H**) CD4 $T_{eff}$ cells isolated from LNs (cervical, axillary, brachial) and lungs of convalescent mice were co-cultured ($0.25x10^{5}$ cells/well) with Treg cell lines ($0.25x10^{5}$, $1.25x10^{5}$, $2.5x10^{5}$ cells/well) and irradiated APCs ($5x10^{5}$ cells/well) and sBp (5 µg/mL). After 4 days IL-17 and IL-10 concentrations in culture supernatants were quantified by ELISA. Data shown are mean ±SD (pooled from 4 individual T cell lines shown in A-G) of triplicate cell culture (technical replicates), representative of two independent experiments.

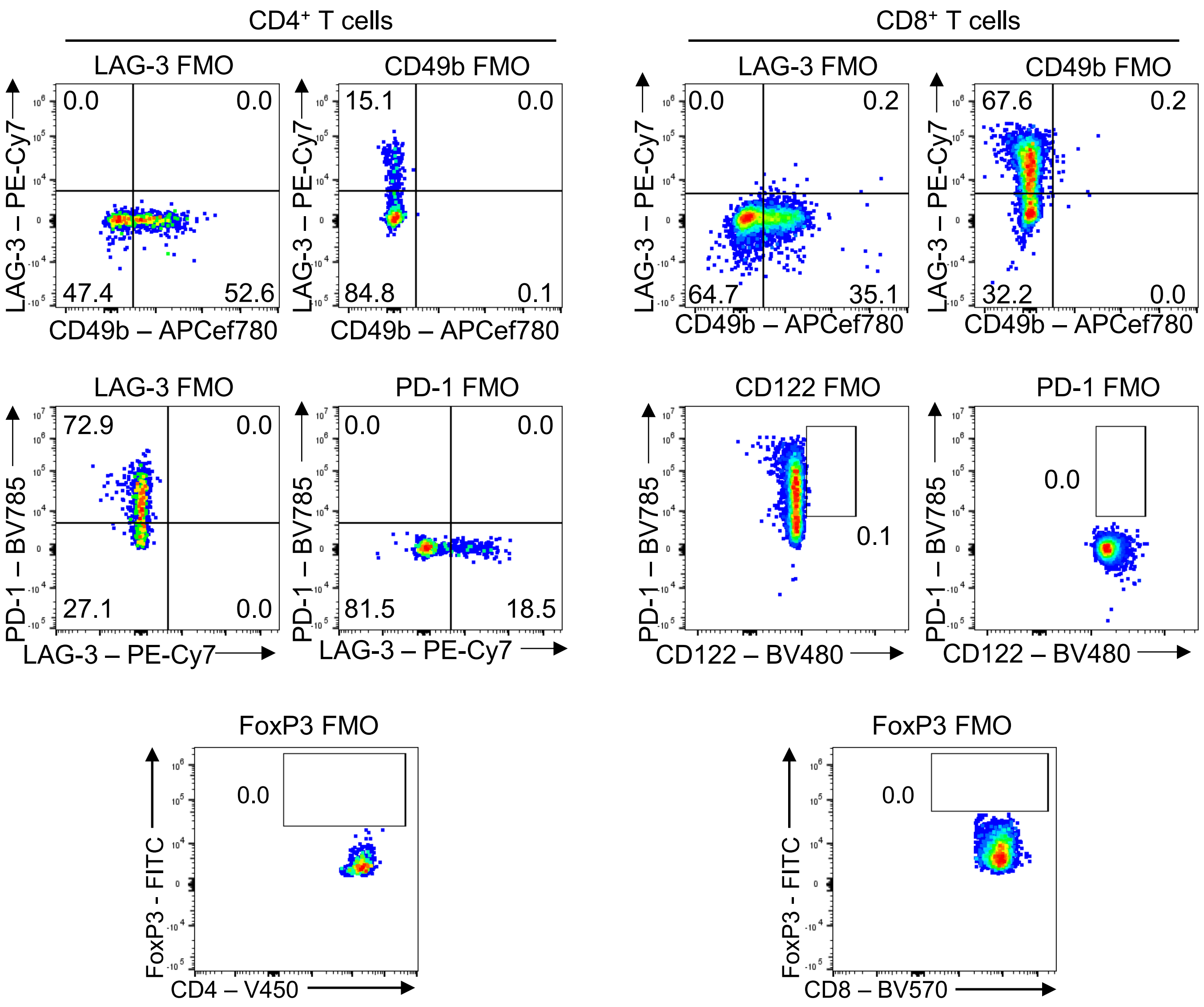


**Figure S7. FMO controls for Treg cells in T cell lines.**

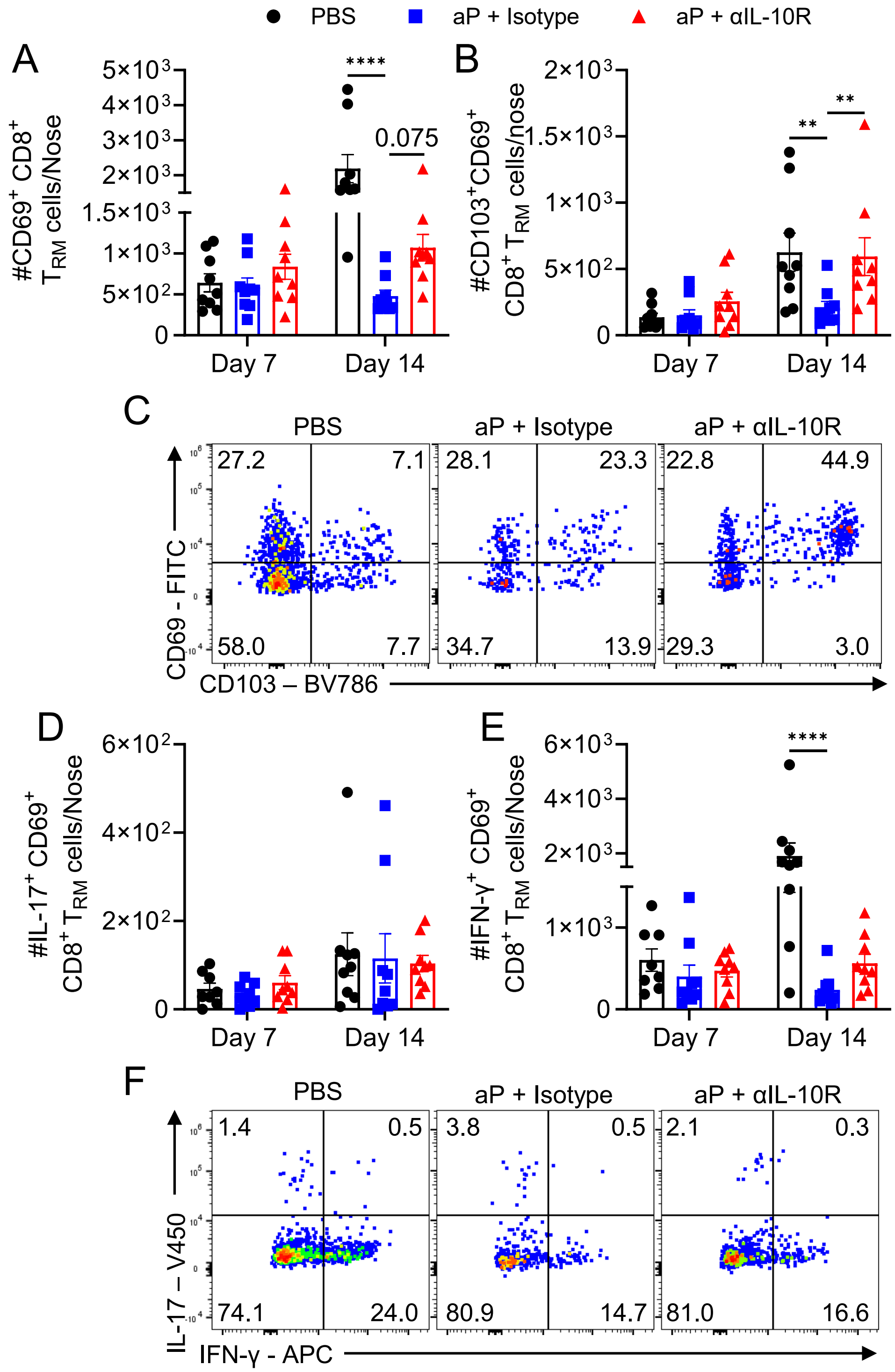


**Figure S8. Blockade of IL-10R at time of *B. pertussis* challenge reverses CD8$^+$ T$_{RM}$ suppression in nasal mucosa in aP-immunized mice.** Mice were immunized i.p. at day 0 and 28 with aP vaccine (Infanrix; 1/50 human dose) or PBS. Mice were aerosol challenged with live *B. pertussis* 2 weeks after boost and were treated i.p. with αIL-10R or isotype control antibody, 1 day and 4 h prior to and on days 4, 8, 12 and 16 after challenge. On days 7 and 14, groups of 4 mice were injected i.v. with αCD45 antibody 10 min prior to euthanasia. Nasal cells were stained with antibodies specific for T$_{RM}$ cell surface markers, or cells were stimulated with PMA and ionomycin for 4 h, prior to ICS and flow cytometric analysis. Absolute numbers of CD69$^+$ (**A**) and CD103$^+$CD69$^+$ (**B**) CD8$^+$ T$_{RM}$ cells, with representative flow cytometry plots for day 14 (**C**). Absolute cell number of IL-17$^+$ (**D**) and IFN-γ$^+$ (**E**) CD8$^+$ T$_{RM}$ cells with representative flow cytometry plots for day 14 (**F**). Data shown are mean ±SEM (n=8-9 mice per group, per time-point), with each symbol representing an individual mouse. All data are pooled from two independent experiments. The control groups in one of the two experiments were shared with those in Figure 4. **p<0.01, ****p<0.0001 by two-way ANOVA with Dunnett's post-test.

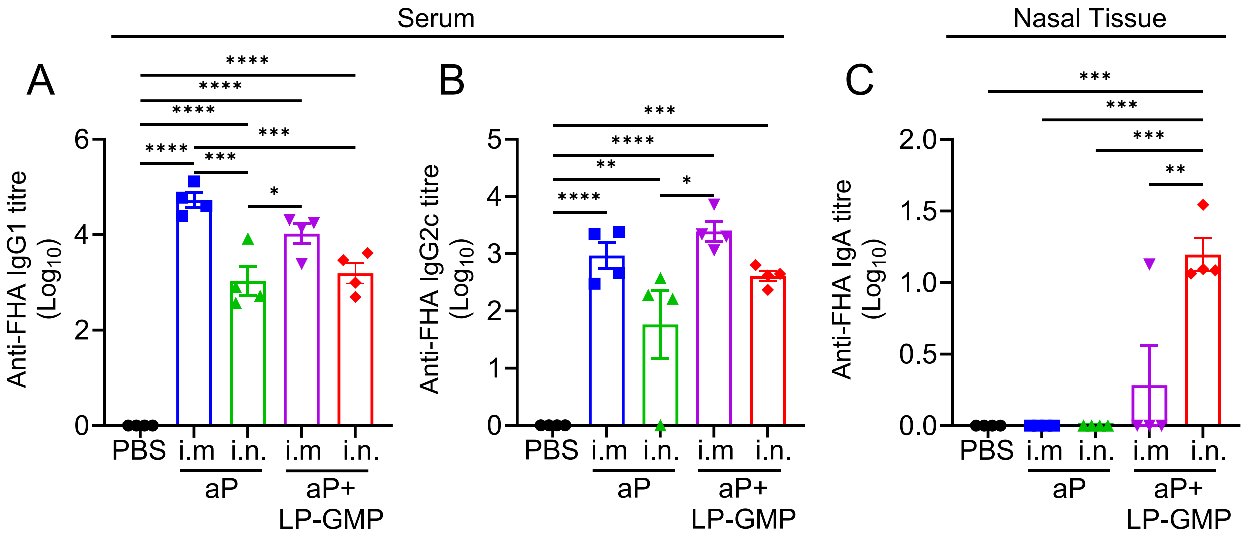


**Figure S9 Addition of LP-GMP to a commercial aP vaccine induces FHA-specific IgA in the nasal mucosa, when delivered by the i.n. route.** Mice were immunized on day 0 and 28 with the aP vaccine (Infanrix; 1/50 human dose) or aP + LP-GMP by i.m. or i.n. route or with PBS as a control. Two weeks following the second immunization, serum and nasal tissue were harvested. FHA-specific antibody concentrations were assessed by ELISA. $Log_{10}$ endpoint titres of serum IgG1 (**A**) and IgG2c (**B**) and IgA in nasal tissue (**C**). Data shown are mean ±SEM (n=4 per group) with each symbol representing an individual mouse. *p<0.05, **p<0.01, ***p<0.001, ****p<0.0001 by one-way ANOVA with Tukey's post-test.

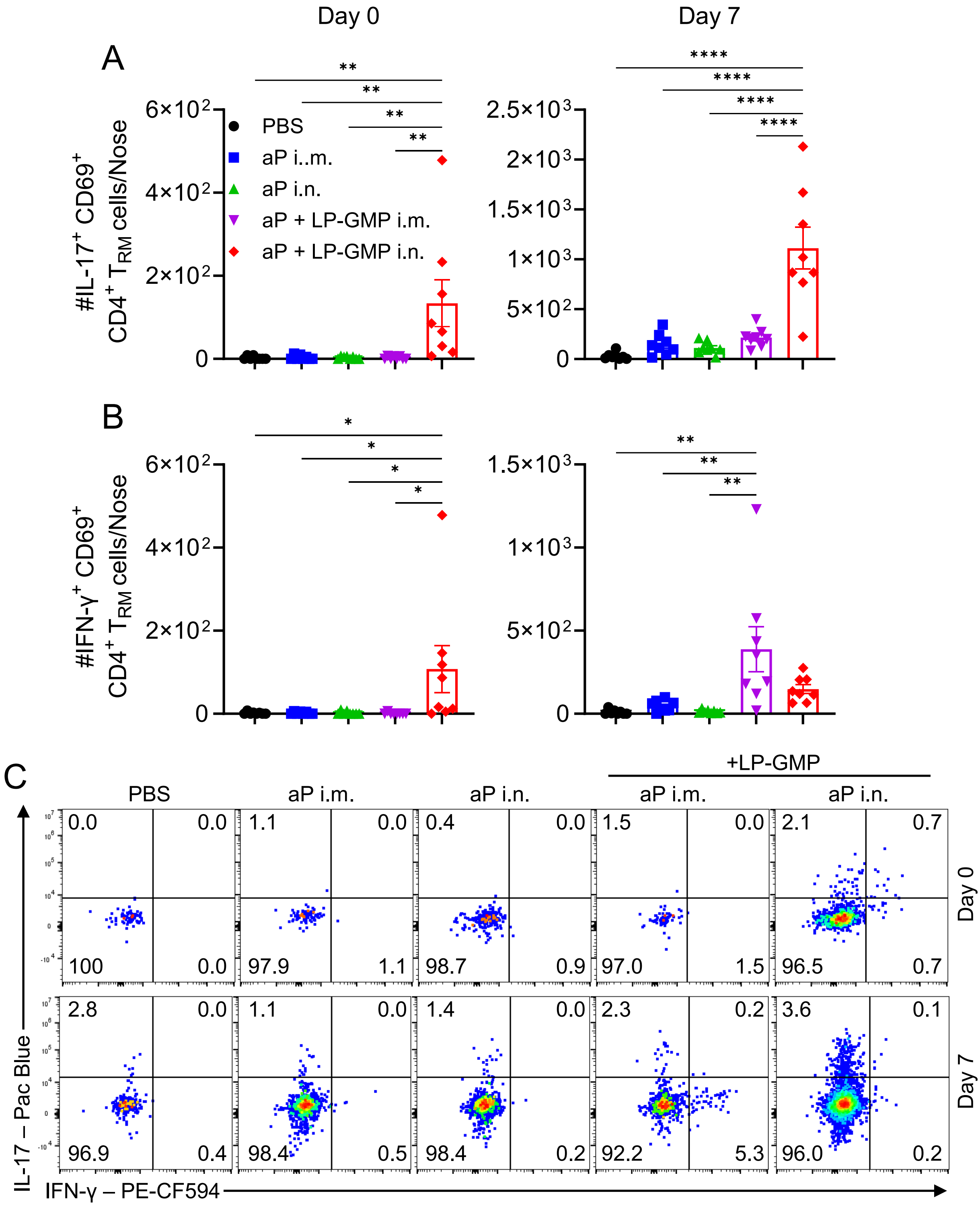
Day 0
Day 7
A
#IL-17+ CD69+ CD4+ TRM cells/Nose
PBS
aP i..m.
aP i.n.
aP + LP-GMP i.m.
aP + LP-GMP i.n.
B
#IFN-γ+ CD69+ CD4+ TRM cells/Nose
C
PBS
aP i.m.
aP i.n.
+LP-GMP
aP i.m.
aP i.n.
Day 0
Day 7
IL-17 – Pac Blue
IFN-γ – PE-CF594

**Figure S10. Addition of LP-GMP to a commercial aP vaccine induces FHA-specific IL-$17^+$ $CD4^+$ $T_{RM}$ cells in the nasal mucosa, when delivered by i.n. route.** Mice were immunized on day 0 and 28 with the aP vaccine (Infanrix; 1/50 human dose) or aP + LP-GMP by i.m. or i.n. route or with PBS as a control. Two weeks following the second immunization, mice were challenged with live *B. pertussis*. On day 0 prior to challenge, and on day 7 post challenge mice were injected i.v. with a fluorochrome-conjugated αCD45 antibody 10 min prior to euthanasia. Single cell suspensions prepared from nasal tissue were stimulated with FHA (2 µg/mL) with anti-CD28 (1 µg/mL) and anti-CD49d (1 µg/mL) for 20 h, with brefeldin A (5 µg/mL) and monensin (1 µg/mL) added for the final 4 h of culture. Cells were stained with antibodies specific for $T_{RM}$ cell surface markers and intracellular cytokines IL-17 and IFN-γ, for assessment by flow cytometry. Mean absolute numbers of FHA-specific IL-$17^+$ (**A**) and IFN-$\gamma^+$ (**B**) $CD69^+CD4^+$ $T_{RM}$ cells on day 0 and day 7 cells, with representative flow cytometry plots (**C**). Data shown are mean ±SEM (n=8 per group), with each symbol representing an individual mouse, and are pooled from two independent experiments. *p<0.05, **p<0.01, ****p<0.0001 by one-way ANOVA with Tukey's post-test.

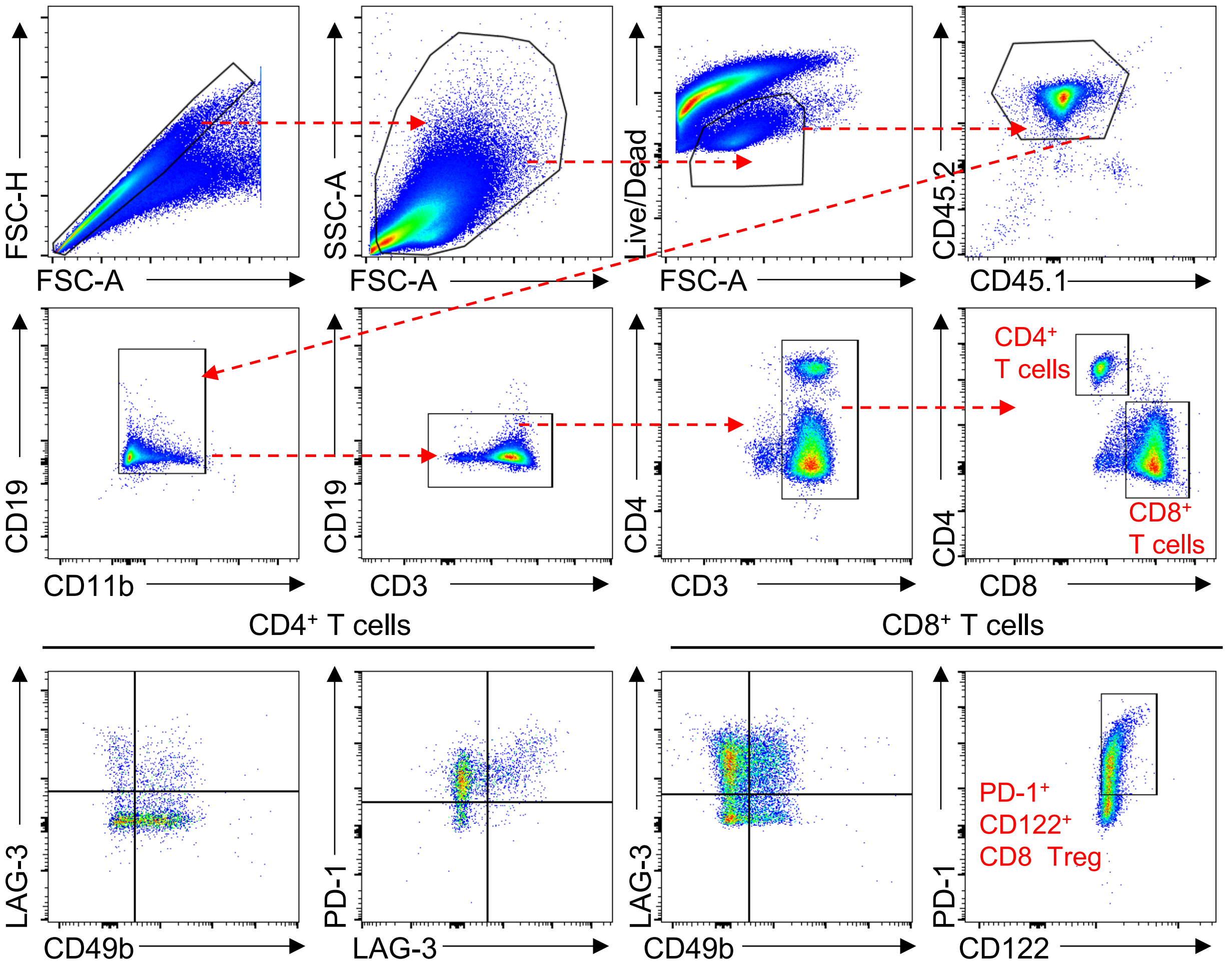


**Figure S11 Flow cytometry gating strategy for Treg cells.** Gating strategy to analyse expression of immune checkpoint molecules and Treg cell markers in FHA- and *B. pertussis*-specific T cell lines.

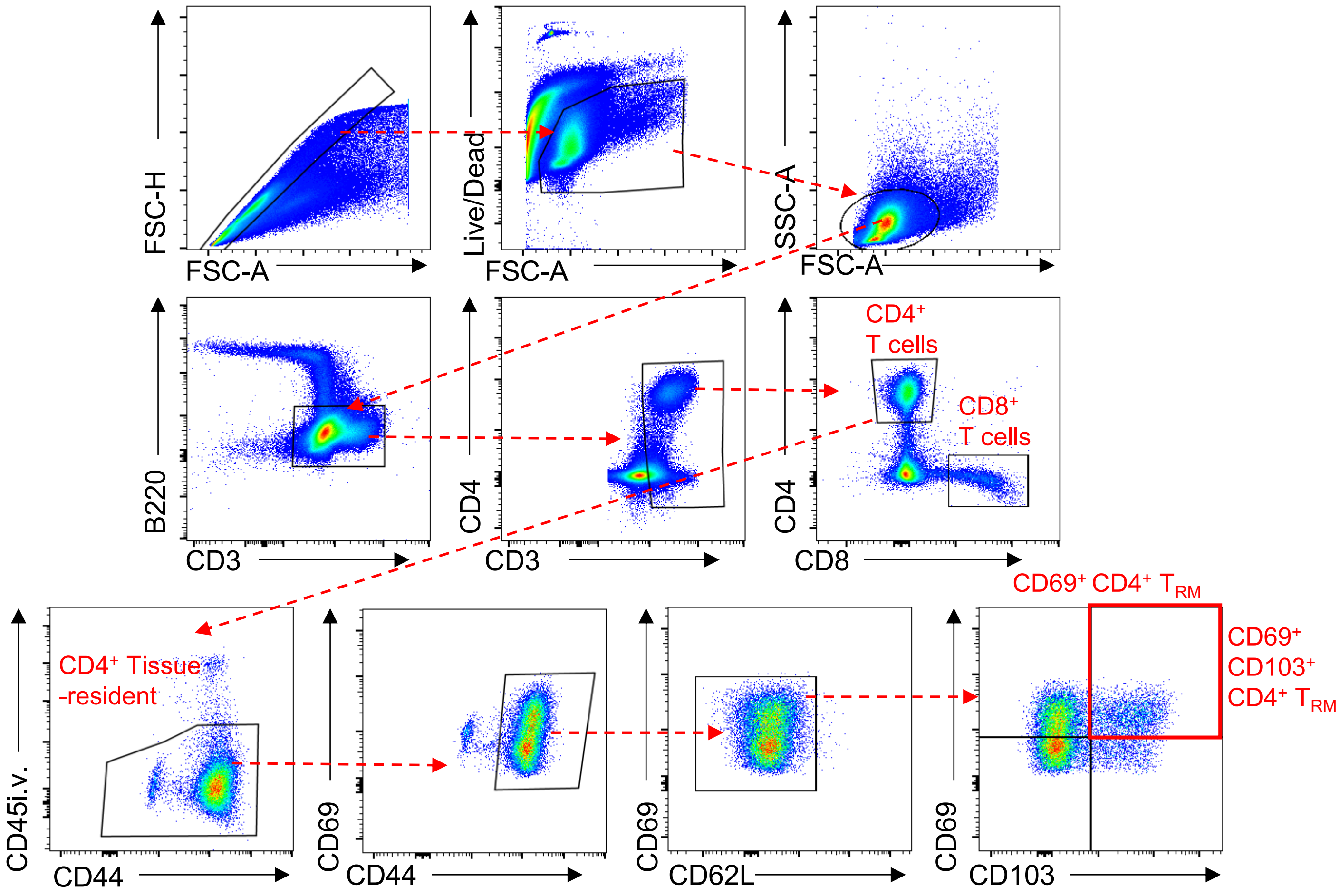


**Figure S12 Flow cytometry gating strategy for CD4$^+$ T$_{RM}$ cells.** Gating strategy for identification of CD4$^+$ T$_{RM}$ cells (CD103$^{\pm}$CD69$^+$CD44$^+$CD62L$^-$CD45i.v.$^-$CD4$^+$CD3$^+$B220$^-$) in the nasal tissue in immunized mice or following *B. pertussis* challenge of immunized mice. In experiments where cells were stimulated with PMA and ionomycin the CD62L staining and gating was omitted.

## Table S1 Antibodies used for flow cytometry

| Antibody | Clone | Source |
|---|---|---|
| Anti-mouse CD3ε - BV650 | 17A2 | BioLegend |
| Anti-mouse CD3ε – BB700 | 145-2C11 | BD Biosciences |
| Anti-mouse CD3ε – APC | 145-2C11 | BioLegend |
| Anti-mouse CD4 – V450 | RM4-5 | BD Biosciences |
| Anti-mouse CD4 - APCef780 | RM4-5 | Invitrogen/eBioscience |
| Anti-mouse CD8 - BV570 | 53-6.7 | BioLegend |
| Anti-mouse CD8 – AF700 | 53-6.7 | Invitrogen/eBioscience |
| Anti-mouse CD11b – PE-Cy5 | M1/70 | Invitrogen/eBioscience |
| Anti-mouse CD19 – BV650 | 1D3 | BD Biosciences |
| Anti-mouse CD44 - BV605 | IM7 | BioLegend |
| Anti-mouse CD45 – PE | 30-F11 | Invitrogen/eBioscience |
| Anti-mouse CD45.1 – AF700 | A20 | BioLegend |
| Anti-mouse CD45.2 – BV711 | 104 | BioLegend |
| Anti-mouse CD45R/B220 – PE-Cy5 | RA3-6B2 | BioLegend |
| Anti-mouse CD45R/B220 – BV650 | RA3-6B2 | BioLegend |
| Anti-mouse CD49b - APCef780 | DX5 | Invitrogen/eBioscience |
| Anti-mouse CD62L - PE-CF594 | MEL-14 | BD Biosciences |
| Anti-mouse CD69 – FITC | H1.2F3 | BioLegend |
| Anti-mouse CD103- BV786 | M290 | BD Biosciences |
| Anti-mouse CD122 – BV480 | 5H4 | BD Biosciences |
| Anti-mouse FoxP3 - FITC | FJK-16s | Invitrogen/eBioscience |
| Anti-mouse LAG-3 – PerCP-ef710 | C9B7W | Invitrogen/eBioscience |
| Anti-mouse LAG-3 – PE-Cy7 | C9B7W | Invitrogen/eBioscience |
| Anti-mouse PD-1 – BV785 | 29F.1A12 | BioLegend |
| Anti-mouse TCRδ - PerCP-ef710 | GL3 | Invitrogen/eBioscience |
| Anti-mouse T-bet – PE-Dazzle594 | 4B10 | Biolegend |
| Anti-mouse RORγt – AF647 | Q31-378 | BD Biosciences |
| Anti-mouse IL-17 – V450 | TC11-18H10 | BD Biosciences |
| Anti-mouse IL-17 – Pacific Blue | TC11-18H10.1 | BioLegend |
| Anti-mouse IFN-γ - APC | XMG1.2 | Invitrogen/eBioscience |
| Anti-mouse IFN-γ - PE-CF594 | XMG1.2 | BD Biosciences |
